# Ground states in the diffusion-dominated regime

**DOI:** 10.1007/s00526-018-1402-2

**Published:** 2018-08-11

**Authors:** José A. Carrillo, Franca Hoffmann, Edoardo Mainini, Bruno Volzone

**Affiliations:** 1 Department of Mathematics, Imperial College London, South Kensington Campus, London, SW7 2AZ UK; 2 Computing & Mathematical Sciences, California Institute of Technology, 1200 E California Boulevard, Pasadena, California, CA 91125 USA; 3 Dipartimento di Ingegneria Meccanica, Università degli Studi di Genova, Piazzale Kennedy, Pad. D, 16129 Genoa, Italy; 4 Dipartimento di Ingegneria, Università degli Studi di Napoli “Parthenope”, 80143 Naples, Italy

**Keywords:** 35K55, 35K65, 49K20

## Abstract

We consider macroscopic descriptions of particles where repulsion is modelled by non-linear power-law diffusion and attraction by a homogeneous singular kernel leading to variants of the Keller–Segel model of chemotaxis. We analyse the regime in which diffusive forces are stronger than attraction between particles, known as the diffusion-dominated regime, and show that all stationary states of the system are radially symmetric non-increasing and compactly supported. The model can be formulated as a gradient flow of a free energy functional for which the overall convexity properties are not known. We show that global minimisers of the free energy always exist. Further, they are radially symmetric, compactly supported, uniformly bounded and $C^\infty$ inside their support. Global minimisers enjoy certain regularity properties if the diffusion is not too slow, and in this case, provide stationary states of the system. In one dimension, stationary states are characterised as optimisers of a functional inequality which establishes equivalence between global minimisers and stationary states, and allows to deduce uniqueness.

## Introduction

We are interested in the diffusion–aggregation equation

1.1 $$\begin{aligned} \partial _t \rho = {\varDelta }\rho ^m +\chi \nabla \cdot \left( \rho \, \nabla S_k[\rho ]\right) \end{aligned}$$

for a density $\rho (t,x)$ of unit mass defined on ${\mathbb {R}}_+ \times {\mathbb {R}}^N$, and where we define the mean-field potential $S_k[\rho ](x) := W_k(x)*\rho (x)$ for some interaction kernel $W_k$. The parameter $\chi >0$ denotes the interaction strength. Since (1.1) conserves mass, is positivity preserving and invariant by translations, we work with solutions $\rho$ in the set

$$\begin{aligned} {{\mathcal {Y}}}:=\left\{ \rho \in L_+^1({\mathbb {R}}^N) \cap L^m({\mathbb {R}}^N)\,,\,||\rho ||_1=1,\, \int _{{\mathbb {R}}^N} x\rho (x)\,dx=0\right\} \, . \end{aligned}$$

The interaction $W_k$ is given by the Riesz kernel

$$\begin{aligned} W_k(x)=\frac{|x|^{k}}{k}, \quad k \in (-N,0). \end{aligned}$$

Let us write $k=2s-N$ with $s\in \left( 0,\frac{N}{2}\right)$. Then the convolution term $S_k[\rho ]$ is governed by a fractional diffusion process,

$$\begin{aligned} c_{N,s}(-{\varDelta })^s S_k[\rho ] = \rho , \quad c_{N,s}=(2s-N) \frac{{\varGamma }\left( \frac{N}{2}-s\right) }{\pi ^{N/2}4^s{\varGamma }(s)} =\frac{k{\varGamma }\left( -k/2\right) }{\pi ^{N/2}2^{k+N}{\varGamma }\left( \frac{k+N}{2}\right) }\, . \end{aligned}$$

For $k>1-N$ the gradient $\nabla S_k[\rho ]:= \nabla \left( W_k *\rho \right)$ is well defined locally. For $k \in \left( -N,1-N\right]$ however, it becomes a singular integral, and we thus define it via a Cauchy principal value,

1.2 $$\begin{aligned} \nabla S_k[\rho ](x) := {\left\{ \begin{array}{ll} \nabla \left( W_k *\rho \right) (x), &{}\quad \text {if} \, \, 1-N<k<0\, , \\ \displaystyle \int _{{\mathbb {R}}^N} \nabla W_k(x-y)\left( \rho (y)-\rho (x)\right) \, dy, &{}\quad \text {if} \, \, -N<k\le 1-N\, . \end{array}\right. } \end{aligned}$$

Here, we are interested in the porous medium case $m>1$ with $N \ge 1$. The corresponding energy functional writes

1.3 $$\begin{aligned} {{\mathcal {F}}}[\rho ]= {{\mathcal {H}}}_m[\rho ]+\chi {{\mathcal {W}}}_k[\rho ] \end{aligned}$$

with

$$\begin{aligned} {{\mathcal {H}}}_m[\rho ]=\frac{1}{m-1} \int _{{\mathbb {R}}^N} \rho ^m(x)\, dx, \quad {{\mathcal {W}}}_k[\rho ]=\frac{1}{2}\iint _{{\mathbb {R}}^N \times {\mathbb {R}}^N} \frac{|x-y|^{k}}{k}\rho (x)\rho (y)\, dxdy\, . \end{aligned}$$

Given $\rho \in {{\mathcal {Y}}}$, we see that ${{\mathcal {H}}}_m$ and ${{\mathcal {W}}}_k$ are homogeneous by taking dilations $\rho ^\lambda (x):=\lambda ^N \rho (\lambda x)$. More precisely, we obtain

$$\begin{aligned} {{\mathcal {F}}}[\rho ^\lambda ]=\lambda ^{N(m-1)}{{\mathcal {H}}}_m[\rho ]+\lambda ^{-k}\chi {{\mathcal {W}}}_k[\rho ]\,. \end{aligned}$$

In other words, the diffusion and aggregation forces are in balance if $N(m-1)=-k$. This is the case for choosing the critical diffusion exponent $m_c:=1-k/N$ called the *fair-competition regime*. In the *diffusion-dominated regime* we choose $m>m_c$, which means that the diffusion part of the functional (1.3) dominates as $\lambda \rightarrow \infty$. In other words, concentrations are not energetically favourable for any value of $\chi >0$ and $m>m_c$. The range $0<m<m_c$ is referred to as the *attraction-dominated regime*. In this work, we focus on the diffusion-dominated regime $m>m_c$.

Further, we define below the diffusion exponent $m^*$ that will play an important role for the regularity properties of global minimisers of ${{\mathcal {F}}}$:

1.4 $$\begin{aligned} m^*:= {\left\{ \begin{array}{ll} \frac{2-k-N}{1-k-N}, &{}\quad \text {if} \quad N\ge 1 \quad \text {and} \quad -N<k<1-N, \\ + \, \infty &{}\quad \text {if} \quad N\ge 2 \quad \text {and} \quad 1-N\le k <0\, . \end{array}\right. } \end{aligned}$$

The main results in this work are summarised in the following two theorems:

### Theorem 1

Let $N\ge 1$, $\chi >0$ and $k \in (-N,0)$. All stationary states of Eq. (1.1) are radially symmetric non-increasing. If $m>m_c$, then there exists a global minimiser $\rho$ of ${{\mathcal {F}}}$ on ${\mathcal {Y}}$. Further, all global minimisers $\rho \in {{\mathcal {Y}}}$ are radially symmetric non-increasing, compactly supported, uniformly bounded and $C^{\infty }$ inside their support. Moreover, all global minimisers of ${{\mathcal {F}}}$ are stationary states of (1.1), according to Definition 1, whenever $m_c<m < m^*$. Finally, if $m_c<m\le 2$, we have $\rho \in {{\mathcal {W}}}^{1,\infty }\left( {\mathbb {R}}^N\right)$.

### Theorem 2

Let $N=1$, $\chi >0$, $k \in (-1,0)$ and $m>m_c$. All stationary states of (1.1) are global minimisers of the energy functional ${{\mathcal {F}}}$ on ${{\mathcal {Y}}}$. Further, stationary states of (1.1) in ${{\mathcal {Y}}}$ are unique.

Diffusion-aggregation at the top equations of the form (1.1) are ubiquitous as macroscopic models of cell motility due to cell adhesion and/or chemotaxis phenomena while taking into account volume filling constraints [10, 29, 45]. The non-linear diffusion models the very strong localised repulsion between cells while the attractive non-local term models either cell movement toward chemosubstance sources or attractive interaction between cells due to cell adhension by long filipodia. They encounter applications in cancer invasion models, organogenesis and pattern formation [18, 24, 28, 42, 46].

The archetypical example of the Keller–Segel model in two dimensions corresponding to the logarithmic case $(m = 1,k=0)$ has been deeply studied by many authors [2, 3, 5, 6, 15, 19, 23, 30–32, 43, 44, 47], although there are still plenty of open problems. In this case, there is an interesting dichotomy based on a critical parameter $\chi _c>0$: the density exists globally in time if $0<\chi <\chi _c$ (diffusion overcomes self-attraction) and expands self-similarly [14, 27], whereas blow-up occurs in finite time when $\chi >\chi _c$ (self-attraction overwhelms diffusion), while for $\chi =\chi _c$ infinitely many stationary solutions exist with intricated basins of attraction [3]. The three-dimensional configuration with Newtonian interaction $(m = 1,k = 2-N)$ appears in gravitational physics [20, 21], although it does not have this dichotomy, belonging to the attraction-dominated regime. However, the dichotomy does happen for the particular exponent $m=4/3$ of the non-linear diffusion for the 3D Newtonian potential as discovered in [4]. This was subsequently generalised for the fair-competition regime where $m=m_c$ for a given $k\in (-N,0)$ in [12, 13].

**Fig. 1:**
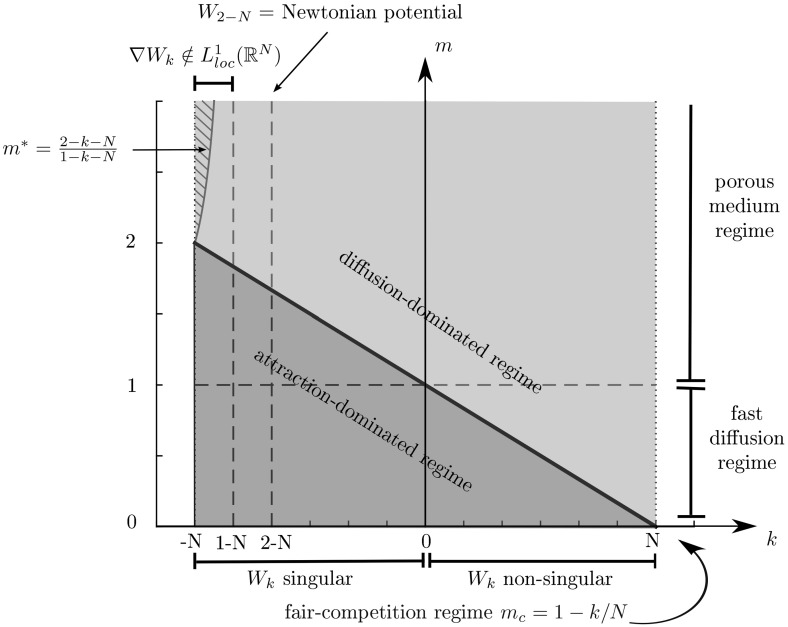
Overview of the parameter space (*k*, *m*) for $N\ge 3$: fair-competition regime ($m=m_c$, red line), diffusion-dominated regime ($m>m_c$, yellow region) and attraction-dominated regime ($m<m_c$, blue region). For $m=m_c$, attractive and repulsive forces are in balance (i.e. in *fair-competition*). For $m_c<m<m^*$ in the diffusion-dominated regime, global minimisers of ${{\mathcal {F}}}$ are stationary states of (1.1), see Theorem 1, a result which we are not able to show for $m\ge m^*$ (striped region)

In fact, as mentioned before two other different regimes appear: the diffusion-dominated case when $m>m_c$ and the attraction-dominated case when $m<m_c$. In Figure 1, we make a sketch of the different regimes including cases related to non-singular kernels for the sake of completeness. Note that non-singular kernels $k>0$ allow for values of $m<1$ corresponding to fast-diffusion behaviour in the diffusion-dominated regime $m>m_c$. We refer to [12, 13] and the references therein for a full discussion of the state of the art in these regimes.

In the diffusion-dominated case, it was already proven in [16] that global minimisers exist in the particular case of $m>1=m_c$ for the logarithmic interaction kernel $k=0$. Their uniqueness up to translation and mass normalisation is a consequence of the important symmetrisation result in [17] asserting that all stationary states to (1.1) for $2-N\le k<0$ are radially symmetric. We will generalise this result to our present framework for the range $-N<k<2-N$ not included in [17] due to the special treatment needed for the arising singular integral terms. This is the main goal of Sect. 2 where we remind the reader the precise definition and basic properties of stationary states for (1.1). In short, we show that stationary solutions are continuous compactly supported radially non-increasing functions with respect to their centre of mass. Some of these results are in fact generalisations of previous results in [12, 17] and we skip some of the details.

Let us finally comment that the symmetrisation result reduces the uniqueness of stationary states to uniqueness of radial stationary states that eventually leads to a full equivalence between stationary states and global minimisers of the free energy (1.3). This was used in [17] to solve completely the 2D case with $m>1=m_c$ for the logarithmic interaction kernel $k=0$, and it was the new ingredient to fully characterise the long-time asymptotics of (1.1) in that particular case.

In view of the main results already announced above, we show in Sect. 3 the existence of global minimisers for the full range $m>m_c$ and $k\in (-N,0)$ which are steady states of the Eq. (1.1) as soon as $m<m^*$. This additional constraint on the range of non-linearities appears only in the most singular range $-N<k<1-N$ and allows us to get the right Hölder regularity on the minimisers in order to make sense of the singular integral in the gradient of the attractive non-local potential force (1.2).

Besides existence of minimisers, Sect. 3 contains some of the main novelties of this paper. First, in order to prove boundedness of minimisers, we develop a fine estimate on the interaction term based on the asymptotics of the Riesz potential of radial functions, and show that this estimate is well suited exactly for the diffusion dominated regime (see Lemma 2 and Theorem 7). Moreover, thanks to the Schauder estimates for the fractional Laplacian, we improve the regularity results for minimisers in [12] and show that they are smooth inside their support, see Theorem 10. This result applies both to the diffusion-dominated and fair-competition regime.

These global minimisers are candidates to play an important role in the long-time asymptotics of (1.1). We show their uniqueness in one dimension by optimal transportation techniques in Sect. 4. The challenging open problems remaining are uniqueness of radially non-increasing stationary solutions to (1.1) in its full generality and the long-time asymptotics of (1.1) in the whole diffusion-dominated regime, even for non-singular kernels within the fast diffusion case.

Plan of the paper: In Sect. 2 we define and analyse stationary states, showing that they are radially symmetric and compactly supported. Section 3 is devoted to global minimisers. We show that global minimisers exist, are bounded and we provide their regularity properties. Eventually, Sect. 4 proves uniqueness of stationary states in the one-dimensional case.

## Stationary states

Let us define precisely the notion of stationary states to the diffusion–aggregation equation (1.1).

### Definition 1

Given ${\bar{\rho }} \in L_+^1\left( {\mathbb {R}}^N\right) \cap L^\infty \left( {\mathbb {R}}^N\right)$ with $||{\bar{\rho }}||_1=1$ and letting ${\bar{S}}_k[{\bar{\rho }}]=W_k*{\bar{\rho }}$, we say that ${\bar{\rho }}$ is a **stationary state** for the evolution equation (1.1) if ${\bar{\rho }}^{m} \in {{\mathcal {W}}}_{loc}^{1,2}\left( {\mathbb {R}}^N\right)$, $\nabla {\bar{S}}_k[{\bar{\rho }}]\in L^1_{loc}\left( {\mathbb {R}}^N\right)$, and it satisfies

2.1 $$\begin{aligned} \nabla {\bar{\rho }}^m=- \chi \, {\bar{\rho }} \nabla {\bar{S}}_k[{\bar{\rho }}] \end{aligned}$$

in the sense of distributions in ${\mathbb {R}}^N$. If $-N<k\le 1-N$, we further require ${\bar{\rho }} \in C^{0,\alpha }\left( {\mathbb {R}}^N\right)$ for some $\alpha \in (1-k-N,1)$.

In fact, as shown in [12] via a near-far field decomposition argument of the drift term, the function $S_k[\rho ]$ and its gradient defined in (1.2) satisfy even more than the regularity $\nabla S_k[\rho ] \in L_{loc}^1\left( {\mathbb {R}}^N\right)$ required in Definition 1:

### Lemma 1

Let $\rho \in L_+^1\left( {\mathbb {R}}^N\right) \cap L^\infty \left( {\mathbb {R}}^N\right)$ with $||\rho ||_1=1$ and $k \in (-N,0)$. Then the following regularity properties hold:

(i) $$ S_k[\rho ] \in L^{\infty }\left( {\mathbb {R}}^N\right) $$.
(ii) $\nabla S_k[\rho ] \in L^{\infty }\left( {\mathbb {R}}^N\right)$, assuming additionally $\rho \in C^{0,\alpha }\left( {\mathbb {R}}^N\right)$ with $\alpha \in (1-k-N,1)$ in the range $k \in (-N,1-N]$.

Lemma 1 implies further regularity properties for stationary states of (1.1). For precise proofs, see [12].

### Proposition 1

Let $k \in (-N,0)$ and $m>m_c$. If ${\bar{\rho }}$ is a stationary state of Eq. (1.1) and $S_k[{\bar{\rho }}]=W_k*{\bar{\rho }}$, then ${\bar{\rho }}$ is continuous on ${\mathbb {R}}^N$, ${\bar{\rho }}^{m-1} \in {{\mathcal {W}}}^{1,\infty }\left( {\mathbb {R}}^N\right)$, and

2.2 $$\begin{aligned} {\bar{\rho }}(x)^{m-1} = \frac{m-1}{m} \left( C[{\bar{\rho }}](x)- \chi S_k[{\bar{\rho }}](x)\right) _+, \quad \forall \, x \in {\mathbb {R}}^N, \end{aligned}$$

where $C[{\bar{\rho }}](x)$ is constant on each connected component of $\mathrm{supp\ }({\bar{\rho }})$.

It follows from Proposition 1 that ${\bar{\rho }} \in {{\mathcal {W}}}^{1,\infty }\left( {\mathbb {R}}^N\right)$ in the case $m_c<m\le 2$.

### Radial symmetry of stationary states

The aim of this section is to prove that stationary states of (1.1) are radially symmetric. This is one of the main results of [17], and is achieved there under the assumption that the interaction kernel is not more singular than the Newtonian potential close to the origin. As we will briefly describe in the proof of the next result, the main arguments continue to hold even for the more singular Riesz kernels $W_{k}$.

#### Theorem 3

(Radiality of stationary states) Let $\chi >0$ and $m>m_c$. If ${\bar{\rho }} \in L^1_+({\mathbb {R}}^N) \cap L^\infty ({\mathbb {R}}^N)$ with $\Vert {\bar{\rho }}\Vert _1=1$ is a stationary state of (1.1) in the sense of Definition 1, then ${\bar{\rho }}$ is radially symmetric non-increasing up to a translation.

#### Proof

The proof is based on a contradiction argument, being an adaptation of that in [17, Theorem 2.2], to which we address the reader the more technical details. Assume that ${\bar{\rho }}$ is *not* radially non-increasing up to *any* translation. By Proposition 1, we have

2.3 $$\begin{aligned} \left| \nabla {\bar{\rho }}^{m-1}(x)\right| \le c \end{aligned}$$

for some positive constant *c* in $\text {supp}({\bar{\rho }})$. Let us now introduce the *continuous Steiner symmetrisation* $S^\tau {\bar{\rho }}$ in direction $e_1 = (1,0,\cdots ,0)$ of ${\bar{\rho }}$ as follows. For any $x_1 \in {\mathbb {R}}, x'\in {\mathbb {R}}^{N-1}, h>0$, let

$$\begin{aligned} S^\tau {\bar{\rho }}(x_1, x') := \int _0^\infty {\mathbb {1}}_{M^\tau (U_{x'}^h)}(x_1) \,dh\,, \end{aligned}$$

where

$$\begin{aligned} U_{x'}^h = \{x_1 \in {\mathbb {R}}: {\bar{\rho }}(x_1, x')>h\} \end{aligned}$$

and $M^\tau (U_{x'}^h)$ is the continuous Steiner symmetrisation of the $U_{x'}^h$ (see [17] for the precise definitions and all the related properties). As in [17], our aim is to show that there exists a continuous family of functions $\mu (\tau ,x)$ such that $\mu (0,\cdot )={\bar{\rho }}$ and some positive constants $C_{1}>0$, $c_{0}>0$ and a small $\delta _{0}>0$ such that the following estimates hold for all $\tau \in [0,\delta _{0}]$:

2.4 $$\begin{aligned}&{{\mathcal {F}}}[\mu (\tau , \cdot )]-{{\mathcal {F}}}[{\bar{\rho }}]\le -c_{0}\tau \end{aligned}$$

2.5 $$\begin{aligned}&|\mu (\tau ,x)-{\bar{\rho }}(x)|\le C_{1}\bar{\rho }(x)\tau \quad \quad \text {for all } x\in {\mathbb {R}}^{N} \end{aligned}$$

2.6 $$\begin{aligned}&\int _{{\varOmega }_{i}}\left( \mu (\tau ,x)-{\bar{\rho }}(x)\right) dx=0\quad \quad \text {for any connected component } {\varOmega }_{i} \text{ of } \text {supp}({\bar{\rho }}). \end{aligned}$$

Following the arguments of the proof in [17, Proposition 2.7], if we want to construct a continuous family $\mu (\tau ,\cdot )$ for (2.5) to hold, it is convenient to modify suitably the continuous Steiner symmetrisation $S^\tau {\bar{\rho }}$ in order to have a better control of the speed in which the level sets $U_{x'}^h$ are moving. More precisely, we define $\mu (\tau ,\cdot ) = {\tilde{S}}^\tau {\bar{\rho }}$ as

$$\begin{aligned} {\tilde{S}}^\tau {\bar{\rho }}_0(x_1, x') := \int _0^\infty {\mathbb {1}}_{M^{v(h)\tau }(U_{x'}^h)}(x_1)\, dh \end{aligned}$$

with *v*(*h*) defined as

$$\begin{aligned} v(h) := {\left\{ \begin{array}{ll} 1 &{}\quad h> h_0\,,\\ 0 &{}\quad 0<h\le h_0\,, \end{array}\right. } \end{aligned}$$

for some sufficiently small constant $h_0>0$ to be determined. Note that this choice of the velocity is different to the one in [17, Proposition 2.7] since we are actually keeping the level sets of ${\tilde{S}}^\tau {\bar{\rho }}(\cdot ,x^{\prime })$ frozen below the layer at height $h_{0}$. Next, we note that inequality (2.3) and the Lipschitz regularity of ${\bar{S}}_{k}$ (Lemma 1) are the only basic ingredients used in the proof of [17, Proposition 2.7] to show that the family $\mu (\tau ,\cdot )$ satisfies (2.5) and (2.6). Therefore, it remains to prove (2.4). Since different level sets of ${\tilde{S}}^\tau {\bar{\rho }}(\cdot ,x^{\prime })$ are moving at different speeds *v*(*h*), we do not have $M^{v(h_1)\tau }(U_{x'}^{h_1}) \subset M^{v(h_2)\tau }(U_{x'}^{h_2})$ for all $h_1>h_2$, but it is still possible to prove that (see [17, Proposition 2.7])

$$\begin{aligned} {{\mathcal {H}}}_m[{\tilde{S}}^\tau {\bar{\rho }}] \le {{\mathcal {H}}}_m[{\bar{\rho }}] \text { for all }\tau \ge 0. \end{aligned}$$

Then, in order to establish (2.4), it is enough to show

2.7 $$\begin{aligned} {{\mathcal {W}}}_k[{\tilde{S}}^\tau {\bar{\rho }}] \le {{\mathcal {W}}}_k[{\bar{\rho }}] -\chi c_0 \tau \quad \text { for all} \, \tau \in [0,\delta _0], \text { for some}\, c_0>0 \, \text {and} \, \delta _0>0. \end{aligned}$$

As in the proof of [17, Proposition 2.7], proving (2.7) reduces to show that for sufficiently small $h_0>0$ one has

2.8 $$\begin{aligned} \left| {{\mathcal {W}}}_k[{\tilde{S}}^\tau {\bar{\rho }}] - {{\mathcal {W}}}_k[ S^\tau {\bar{\rho }}]\right| \le \frac{c\chi \tau }{2}\quad \text { for all} \, \tau . \end{aligned}$$

To this aim, we write

$$\begin{aligned} S^\tau {\bar{\rho }}(x_1, x') = \int _{h_0}^\infty {\mathbb {1}}_{M^\tau (U_{x'}^h)}(x_1)dh + \int _{0}^{h_0} {\mathbb {1}}_{M^\tau (U_{x'}^h)}(x_1)dh =: f_1(\tau ,x)+f_2(\tau ,x) \end{aligned}$$

and we split ${\tilde{S}}^\tau {\bar{\rho }}$ similarly, taking into account that $v(h)=1$ for all $h>h_0$:

$$\begin{aligned} {\tilde{S}}^\tau {\bar{\rho }}(x_1, x') = f_1(\tau ,x) + \int _{0}^{h_0} {\mathbb {1}}_{M^{v(h)\tau }(U_{x'}^h)}(x_1)dh =: f_1(\tau ,x) + {\tilde{f}}_2(\tau ,x). \end{aligned}$$

Note that

$$\begin{aligned} f_{2}=S^{\tau }({\mathcal {T}}^{h_{0}}{\bar{\rho }}), \end{aligned}$$

where ${\mathcal {T}}^{h_{0}}{\bar{\rho }}$ is the truncation at height $h_{0}$ of ${\bar{\rho }}$. Since $v(h)=0$ for $h\le h_{0}$, we have

$$\begin{aligned} {\tilde{f}}_2={\mathcal {T}}^{h_{0}}{\bar{\rho }}. \end{aligned}$$

If we are in the singular range $k\in (-N,1-N]$, we have ${\bar{\rho }} \in C^{0,\alpha }\left( {\mathbb {R}}^N\right)$ for some $\alpha \in (1-k-N,1)$. Since the continuous Steiner symmetrisation decreases the modulus of continuity (see [8, Theorem 3.3] and [8, Corollary 3.1]), we also have $S^\tau {\bar{\rho }},\, f_{2},\,{\tilde{f}}_2\in C^{0,\alpha }\left( {\mathbb {R}}^N\right)$. Further, Lemma 1 and the arguments of [17, Proposition 2.7] guarantee that the expressions

$$\begin{aligned}&A_1(\tau ):=\left| \int f_2 (W_k *f_1)-{\tilde{f}}_2 (W_k *f_1)dx\right| \quad \text {and} \\&A_2(\tau ):=\left| \int f_2 (W_k *f_2)-{\tilde{f}}_2 (W_k *{\tilde{f}}_2)dx\right| \end{aligned}$$

can be controlled by $||{\bar{\rho }}||_\infty$ and the $\alpha$-Hölder seminorm of ${\bar{\rho }}$. Hence, we can apply the argument in [17, Proposition 2.7] to conclude for the estimate (2.8). Now it is possible to proceed exactly as in the proof of [17, Theorem 2.2] to show that for some positive constant $C_{2}$, we have the quadratic estimate

$$\begin{aligned} \left| {{\mathcal {F}}}[\mu (\tau ,\cdot )]-{{\mathcal {F}}}[{\bar{\rho }}]\right| \le C_{2}\tau ^{2}, \end{aligned}$$

which is a contradiction with (2.4) for small $\tau$. $\square$

### Stationary states are compactly supported

In this section, we will prove that all stationary states of Eq. (1.1) have compact support, which agrees with the properties shown in [16, 17, 33]. We begin by stating a useful asymptotic estimate on the Riesz potential inspired by [50, § 4]. For the proof of Proposition 2, see Appendix 1.

#### Proposition 2

(Riesz potential estimates) Let $k \in (-N,0)$ and let $\rho \in {{\mathcal {Y}}}$ be radially symmetric.

(i) If $1-N<k<0$, then $|x|^k*\rho (x)\le C_1 |x|^{k}$ on ${\mathbb {R}}^N$.
(ii) If $-N<k\le 1-N$ and if $\rho$ is supported on a ball $B_R$ for some $R<\infty$, then
  $$\begin{aligned} |x|^k*\rho (x)\le C_2 T_k(|x|,R)\, |x|^{k}, \quad \forall \, \, |x|>R, \end{aligned}$$
  where
  2.9 $$\begin{aligned} T_k(|x|,R):=\left\{ \begin{array}{l@{\quad }l} \left( \frac{|x|+R}{|x|-R}\right) ^{1-k-N}&{}\text{ if } \, k\in (-N,1-N),\\ \\ \left( 1+\log \left( \frac{|x|+R}{|x|-R}\right) \right) &{}\text{ if }\, k=1-N \end{array}\right. \end{aligned}$$

Here, $C_1>0$ and $C_2>0$ are explicit constants depending only on *k* and *N*.

From the above estimate, we can derive the expected asymptotic behaviour at infinity.

#### Corollary 1

Let $\rho \in {{\mathcal {Y}}}$ be radially non-increasing. Then $W_k*\rho$ vanishes at infinity, with decay not faster than that of $|x|^{k}$.

#### Proof

Notice that Proposition 2(i) entails the decay of the Riesz potential at infinity for $1-N<k<0$. Instead, let $-N<k\le 1-N$. Let $r\in (1-k-N,1)$ and notice that $|y|^{k}\le |y|^{k+r}$ if $|y|\ge 1$, so that if $B_1$ is the unit ball centered at the origin we have

$$\begin{aligned} \begin{aligned} |x|^k*\rho (x)&\le \int _{B_{1}}\rho (x-y)|y|^{k}\,dy+\int _{B_1^C}\rho (x-y)|y|^{k+r}\,dy\\&\le \left( \sup _{y\in B_1}\rho (x-y)\right) \int _{B_1}|y|^{k}\,dy+(W_{k+r}*\rho )(x). \end{aligned} \end{aligned}$$

The first term in the right hand side vanishes as $|x|\rightarrow \infty$, since $y\mapsto |y|^{k}$ is integrable at the origin, and since $\rho$ is radially non-increasing and vanishing at infinity as well. The second term goes to zero at infinity thanks to Proposition 2(i), since the choice of *r* yields $k+r>1-N$.

On the other hand, the decay at infinity of the Riesz potential can not be faster than that of $|x|^{k}$. To see this, notice that there holds

$$\begin{aligned} |x|^k*\rho (x)\ge \int _{B_1}\rho (y)|x-y|^{k}\,dy \ge (|x|+1)^{k} \int _{B_1}\rho (y)\,dy \end{aligned}$$

with $\int _{B_1}\rho >0$ since $\rho \in {{\mathcal {Y}}}$ is radially non-increasing. $\square$

As a rather simple consequence of Corollary 1, we obtain:

#### Corollary 2

Let ${\bar{\rho }}$ be a stationary state of (1.1). Then ${\bar{\rho }}$ is compactly supported.

#### Proof

By Theorem 3 we have that ${\bar{\rho }}$ is radially non-increasing up to a translation. Since the translation of a stationary state is itself a stationary state, we may assume that ${\bar{\rho }}$ is radially symmetric with respect to the origin. Suppose by contradiction that ${\bar{\rho }}$ is supported on the whole of ${\mathbb {R}}^N$, so that Eq. (2.2) holds on the whole ${\mathbb {R}}^N$, with $C_{k}[{\bar{\rho }}](x)$ replaced by a unique constant *C*. Then we necessarily have $C=0$. Indeed, ${\bar{\rho }}^{m-1}$ vanishes at infinity since it is radially non-increasing and integrable, and by Corollary 1 we have that $S_{k}[{\bar{\rho }}]=W_k*{\bar{\rho }}$ vanishes at infinity as well. Therefore

$$\begin{aligned} {\bar{\rho }}=\left( \frac{\chi (m-1)}{m}S_{k}[{\bar{\rho }}]\right) ^{1/(m-1)}. \end{aligned}$$

But Corollary 1 shows that $W_k*\rho$ decays at infinity not faster than $|x|^{k}$ and this would entail, since $m>m_c$, a decay at infinity of $\rho$ not faster than that of $|x|^{-N}$, contradicting the integrability of $\rho$. $\square$

## Global minimisers

We start this section by recalling a key ingredient for the analysis of the regularity of the drift term in (1.1), i.e. certain functional inequalities which are variants of the Hardy-Littlewood-Sobolev (HLS) inequality, also known as the weak Young’s inequality [36, Theorem 4.3]: for all $f \in L^p({\mathbb {R}}^N)$, $g \in L^q({\mathbb {R}}^N)$ there exists an optimal constant $C_{HLS}=\mathrm{depends\, on\, N\, too}>0$ such that

3.1 $$\begin{aligned}&\left| \iint _{{\mathbb {R}}^N\times {\mathbb {R}}^N} f(x) {|x-y|^{k}} g(y)\, dxdy\right| \le C_{HLS} \Vert f\Vert _{p} \Vert g\Vert _{q}, \nonumber \\&\quad \text {if}\quad \dfrac{1}{p} + \dfrac{1}{q} = 2 + \dfrac{k}{N} \, , \quad p,q>1,\quad k\in (-N,0) \, . \end{aligned}$$

The optimal constant $C_{HLS}$ is found in [35]. In the sequel, we will make use of the following variations of the above HLS inequality:

### Theorem 4

Let $k \in (-N,0)$, and $m>m_c$. For $f \in L^1({\mathbb {R}}^N) \cap L^m({\mathbb {R}}^N)$, we have

3.2 $$\begin{aligned} \left| \iint _{{\mathbb {R}}^N\times {\mathbb {R}}^N} {|x-y|^{k}}f(x)f(y) dxdy\right| \le C_* ||f||^{(k+N)/N}_1 ||f||^{m_c}_{m_c}, \end{aligned}$$

where $C_*=C_*(k,m,N)$ is the best constant.

### Proof

The inequality is a direct consequence of the standard sharp HLS inequality and of Hölder’s inequality. It follows that $C_*$ is finite and bounded from above by the optimal constant in the HLS inequality. $\square$

### Existence of global minimisers

#### Theorem 5

(Existence of global minimisers) For all $\chi >0$ and $k \in (-N,0)$, there exists a global minimiser $\rho$ of ${{\mathcal {F}}}$ in ${{\mathcal {Y}}}$. Moreover, all global minimisers of ${{\mathcal {F}}}$ in ${{\mathcal {Y}}}$ are radially non-increasing.

We follow the concentration compactness argument as applied in Appendix A.1 of [33]. Our proof is based on [38, Theorem II.1, Corollary II.1]. Let us denote by ${\mathcal {M}}^p({\mathbb {R}}^N)$ the Marcinkiewicz space or weak $L^p$ space.

#### Theorem 6

(see [38, Theorem II.1]) Suppose $W\in {\mathcal {M}}^p({\mathbb {R}}^N)$, $1<p<\infty$, and consider the problem

$$\begin{aligned} I_M = \inf _{\rho \in {{\mathcal {Y}}}_{q,M}} \left\{ \frac{1}{m-1} \int _{{\mathbb {R}}^N} \rho ^m dx + \frac{\chi }{2}\iint _{{\mathbb {R}}^N\times {\mathbb {R}}^N} W(x-y)\rho (x)\rho (y)\,dxdy\right\} \, . \end{aligned}$$

where

$$\begin{aligned} {{\mathcal {Y}}}_{q,M}=\left\{ \rho \in L^q({\mathbb {R}}^N)\cap L^1({\mathbb {R}}^N), \, \rho \ge 0\, \, a.e., \int _{{\mathbb {R}}^N} \rho (x) \, dx=M\right\} , \quad q=\frac{p+1}{p}<m\, . \end{aligned}$$

Then there exists a minimiser of problem $(I_M)$ if the following holds:

3.3 $$\begin{aligned} I_{M_0} < I_{M} + I_{M_0-M} \quad \text {for all} \, \, M \in (0,M_0)\, . \end{aligned}$$

#### Proposition 3

(see [38, Corollary II.1]) Suppose there exists some $\lambda \in (0,N)$ such that

$$\begin{aligned} W(tx) \ge t^{-\lambda }W(x) \end{aligned}$$

for all $t\ge 1$. Then (3.3) holds if and only if

3.4 $$\begin{aligned} I_{M} < 0 \quad \text {for all} \, \,M>0\, . \end{aligned}$$

#### Proof of Theorem 5

First of all, notice that our choice of potential $W_k(x)=|x|^{k}/k$ is indeed in ${\mathcal {M}}^p({\mathbb {R}}^N)$ with $p=-N/k$. Further, it can easily be verified that Proposition 3 applies with $\lambda =-k$. Hence we are left to show that there exists a choice of $\rho \in {{\mathcal {Y}}}_{q,M}$ such that ${{\mathcal {F}}}[\rho ]<0$. Let us fix $R>0$ and define

$$\begin{aligned} \rho _*(x):=\frac{MN}{\sigma _N R^N}\, {\mathbb {1}}_{B_R}(x), \end{aligned}$$

where $B_R$ denotes the ball centered at zero and of radius $R>0$, and where $\sigma _N=2 \pi ^{(N/2)}/{\varGamma }(N/2)$ denotes the surface area of the *N*-dimensional unit ball. Then

$$\begin{aligned} {{\mathcal {H}}}_m[\rho _*]&=\frac{1}{m-1} \int _{{\mathbb {R}}^N} \rho _*^m dx =\frac{(MN)^m \sigma _N^{1-m}}{N(m-1)} \, R^{N(1-m)}, \\ {{\mathcal {W}}}_k[\rho _*]&=\frac{1}{2}\iint _{{\mathbb {R}}^N\times {\mathbb {R}}^N} W_k(x-y)\rho _*(x)\rho _*(y)\, dx dy\\&= \frac{(MN)^2}{2k\sigma _N^2 R^{2N}}\iint _{{\mathbb {R}}^N\times {\mathbb {R}}^N}|x-y|^{k}{\mathbb {1}}_{B_R}(x){\mathbb {1}}_{B_R}(y)\, dx dy \\&\le \frac{(MN)^2}{2k\sigma _N^2 R^{2N}} (2R)^{k} \frac{\sigma _N^2}{N^2}R^{2N} = 2^{k-1}M^2 \frac{R^{k}}{k}<0\, . \end{aligned}$$

We conclude that

$$\begin{aligned} {{\mathcal {F}}}[\rho _*]={{\mathcal {H}}}_m[\rho _*]+\chi {{\mathcal {W}}}_k[\rho _*] \le \frac{M^m N^{m-1} \sigma _N^{1-m}}{(m-1)} \, R^{N(1-m)} +2^{k-1}M^2\chi \frac{R^{k}}{k}\, . \end{aligned}$$

Since we are in the diffusion-dominated regime $N(1-m)< k<0$, we can choose $R>0$ large enough such that ${{\mathcal {F}}}[\rho _*]<0$, and hence condition (3.4) is satisfied. We conclude by Proposition 3 and Theorem 6 that there exists a minimiser ${\bar{\rho }}$ of ${{\mathcal {F}}}$ in ${{\mathcal {Y}}}_{q,M}$ with $q=(p+1)/p=(N-k)/N$.

It can easily be seen that in fact ${\bar{\rho }} \in L^m({\mathbb {R}}^N)$ using the HLS inequality (3.1):

$$\begin{aligned} -{{\mathcal {W}}}_k[\rho ]=\frac{1}{2} \iint _{{\mathbb {R}}^N \times {\mathbb {R}}^N} \frac{|x-y|^{k}}{(-k)}\rho (x)\rho (y)\, dxdy \le \frac{C_{HLS}}{(-2k)} ||\rho ||_{r}^2, \end{aligned}$$

where $r=2N/(2N+k)=2p/(2p-1)$. Using Hölder’s inequality, we find

$$\begin{aligned} -{{\mathcal {W}}}_k[\rho ]\le \frac{C_{HLS}}{(-2k)} ||\rho ||_q^q ||\rho ||_1^{2-q} \, . \end{aligned}$$

Hence, since $${{\mathcal {F}}}[{\bar{\rho }}]<0$$,

$$\begin{aligned} ||{\bar{\rho }}||_m^m\le -\chi (m-1) {{\mathcal {W}}}_k[{\bar{\rho }}] \le \chi (m-1)\left( \frac{M^{2-q}C_{HLS}}{(-2k)}\right) ||{\bar{\rho }}||_q^q < \infty \, . \end{aligned}$$

Translating ${\bar{\rho }}$ so that its centre of mass is at zero and choosing $M=1$, we obtain a minimiser ${\bar{\rho }}$ of ${{\mathcal {F}}}$ in ${{\mathcal {Y}}}$. Moreover, by Riesz’s rearrangement inequality [36, Theorem 3.7], we have

$$\begin{aligned} {{\mathcal {W}}}_k[\rho ^{\#}]\le {{\mathcal {W}}}_k[\rho ], \quad \forall \rho \in {{\mathcal {Y}}}, \end{aligned}$$

where $\rho ^{\#}$ is the Schwarz decreasing rearrangement of $\rho$. Thus, if ${\bar{\rho }}$ is a global minimiser of ${{\mathcal {F}}}$ in ${{\mathcal {Y}}}$, then so is ${\bar{\rho }}^{\#}$, and it follows that

$$\begin{aligned} {{\mathcal {W}}}_k[{\bar{\rho }}^{\#}]= {{\mathcal {W}}}_k[{\bar{\rho }}]\, . \end{aligned}$$

We conclude from [36, Theorem 3.7] that ${\bar{\rho }}={\bar{\rho }}^{\#}$, and so all global minimisers of ${{\mathcal {F}}}$ in ${{\mathcal {Y}}}$ are radially symmetric non-increasing. $\square$

#### Remark 1

An alternative and more direct proof of the existence of global minimisers for ${{\mathcal {F}}}$ can be achieved by a scaling argument along the lines of [4, 12, 37]. More precisely, taking dilations $\rho ^\lambda (x):=\lambda ^N \rho (\lambda x)$ of a given $\rho \in {{\mathcal {Y}}}$, we define $g(\lambda ):={{\mathcal {F}}}[\rho ^\lambda ]$ and $\delta :=N(m-1)+k>0$. Optimising over $\lambda$, we find a unique $\lambda ^*>0$ such that $g'(\lambda ^*)=0$:

$$\begin{aligned} \lambda ^*:=\left( \frac{k\chi {{\mathcal {W}}}_k[\rho ]}{N(m-1){{\mathcal {H}}}_m[\rho ]}\right) ^{1/\delta }\,. \end{aligned}$$

Note that

$$\begin{aligned} g''(\lambda ^*)=\left( 2(k+1)-\delta \right) \left( k-\delta \right) ^{-\frac{k+2}{\delta }} \left( k\chi {{\mathcal {W}}}_k[\rho ]\right) ^{1-\frac{k+2}{\delta }} {{\mathcal {H}}}_m[\rho ]^{\frac{k+2}{\delta }}\, . \end{aligned}$$

Substitution the optimal dilation $\rho ^{\lambda ^*}$ of $\rho$ into the energy functional ${{\mathcal {F}}}$, we obtain

$$\begin{aligned} {{\mathcal {F}}}[\rho ^{\lambda ^*}]=-c_1 {\varLambda }[\rho ], \end{aligned}$$

where

$$\begin{aligned} {\varLambda }[\rho ]:=\left( k\chi {{\mathcal {W}}}_k[\rho ]\right) ^{1-\frac{k}{\delta }} {{\mathcal {H}}}_m[\rho ]^{\frac{k}{\delta }}, \quad c_1:= \frac{\delta }{(-k)(\delta -k)^{1-\frac{k}{\delta }}}>0\, . \end{aligned}$$

The goal is therefore to show existence of ${\bar{\rho }} \in {{\mathcal {Y}}}$ maximising ${\varLambda }[\rho ]$. If such a global maximiser exists, then ${\bar{\rho }}^{\lambda ^*}$ provides a global minimiser of ${{\mathcal {F}}}$ over ${{\mathcal {Y}}}$, and ${{\mathcal {F}}}[\rho ]\ge -c_1{\varLambda }[{\bar{\rho }}]={{\mathcal {F}}}[{\bar{\rho }}^{\lambda ^*}]$. Note that ${\varLambda }[\rho ]$ is invariant by dilations, ${\varLambda }[\rho ^\lambda ]={\varLambda }[\rho ] \, \, \forall \lambda >0$, and we can therefore apply the same strategy as in the existence proof [12, Proposition 3.4].

Global minimisers of ${{\mathcal {F}}}$ satisfy a corresponding Euler–Lagrange condition. The proof can be directly adapted from [16, Theorem 3.1] or [12, Proposition 3.6], and we omit it here.

#### Proposition 4

Let $k \in (-N,0)$ and $m>m_c$. If $\rho$ is a global minimiser of the free energy functional ${{\mathcal {F}}}$ in ${{\mathcal {Y}}}$, then $\rho$ is radially symmetric and non-increasing, satisfying

3.5 $$\begin{aligned} \rho ^{m-1}(x) = \left( \frac{m-1}{m}\right) \,\left( D[\rho ]-\chi S_k[\rho ](x)\right) _+ \quad \text {a.e. in}\, \, {\mathbb {R}}^N. \end{aligned}$$

Here, we denote

$$\begin{aligned} D[\rho ] := 2 {{\mathcal {F}}}[\rho ] + \left( \frac{m-2}{m-1}\right) ||\rho ||_m^m, \quad \rho \in {{\mathcal {Y}}}\,. \end{aligned}$$

### Boundedness of global minimisers

This section is devoted to showing that all global minimisers of ${{\mathcal {F}}}$ in ${{\mathcal {Y}}}$ are uniformly bounded. In the following, for a radial function $\rho \in L^1({\mathbb {R}}^N)$ we denote by $M_\rho (R):=\int _{B_R}\rho \, dx$ the corresponding mass function, where $B_R$ is a ball of radius *R*, centered at the origin. We start with the following technical lemma:

#### Lemma 2

Let $\chi >0$, $-N<k<0$, $m>1$ and $0\le q< m/N$. Assume $\rho \in {{\mathcal {Y}}}$ is radially non-increasing. For a fixed $H>0$, the level set $\{\rho \ge H\}$ is a ball centered at the origin whose radius we denote by $A_H$. Then we have the following cross-range interaction estimate: there exists $H_0>1$, depending only on $q,N,m,\Vert \rho \Vert _m$, such that, for any $H>H_0$,

$$\begin{aligned} \int _{B_{A_H}^C}\int _{B_{A_H}}|x-y|^{k} \rho (x)\rho (y)\,dx\,dy\le C_{k,N}\, M_{\rho }({A_H})\, {\mathcal {K}}_{k,q,N}(H), \end{aligned}$$

where

$$\begin{aligned} {\mathcal {K}}_{k,q,N}(H):=\left\{ \begin{array}{ll} H^{1-q(k+N)}+ H^{-kq} &{}\quad \text{ if } \quad k\in (-N,0),\; k\ne 1-N,\\ H^{1-q}(2+\log (1+H^q))+ H^{q(N-1)} &{}\quad \text{ if } \quad k=1-N \end{array}\right. \end{aligned}$$

and $C_{k,N}$ is a constant depending only on *k* and *N*.

#### Proof

Notice that the result is trivial if $\rho$ is bounded. The interesting case here is $\rho$ unbounded, implying that $A_H>0$ for any $H>0$.

First of all, since $\rho \in L^m({\mathbb {R}}^N)$ and $\rho \ge H$ on $B_{A_H}$, the estimate

$$\begin{aligned} \frac{\sigma _N A_H^N}{N} H^m=\int _{B_{A_H}} H^m\le \int _{B_{A_H}}\rho ^m\le ||\rho ||_m^m \end{aligned}$$

implies that $H^q A_H$ is vanishing as $H\rightarrow +\infty$ as soon as $q<m/N$, and in particular that we can find $H_0>1$, depending only on $q,m,N, ||\rho ||_m$, such that

$$\begin{aligned} H^{-q}\ge 2 A_H\quad \text{ for } \text{ any } \, H>H_0. \end{aligned}$$

We fix $q\in [0,m/N)$ and $H>H_0$ as above from here on.

Let us make use of Proposition 2, which we apply to the compactly supported function $\rho _H:=\rho {\mathbb {1}}_{\{\rho \ge H\}}/M_\rho \left( A_H\right)$.

Case $1-N<k<0$: Proposition 2(i) applied to $\rho _H$ gives the estimate

$$\begin{aligned} \int _{B_{A_H}}|x-y|^{k}\rho (y)\,dy\le C_1 M_\rho \left( A_H\right) |x|^{k} , \quad \forall x\in {\mathbb {R}}^N, \end{aligned}$$

and hence, integrating against $\rho$ on $B_{A_H}^C$ and using $\rho \le H$ on $B^C_{A_H}$,

$$\begin{aligned}&\int _{B_{A_H}^C}\int _{B_{A_H}}|x-y|^{k} \rho (x)\rho (y)\,dx\,dy \le C_1 M_\rho \left( A_H\right) \int _{B_{A_H}^C}|x|^{k} \rho (x)\,dx\\&\quad = C_1 M_\rho \left( A_H\right) \left( \int _{B_{A_H}^C\cap B_{H^{-q}}}|x|^{k} \rho (x)\,dx +\int _{B_{A_H}^C\setminus {B_{H^{-q}}}} |x|^{k} \rho (x)\,dx \right) \\&\quad \le C_1 M_\rho \left( A_H\right) \left( H \int _{B_{A_H}^C\cap B_{H^{-q}}}|x|^k\, dx +H^{-kq}\int _{B_{A_H}^C\setminus {B_{H^{-q}}}} \rho (x)\,dx \right) \\&\quad \le C_1 M_\rho \left( A_H\right) \left( H \sigma _N \int _{A_H}^{H^{-q}}r^{k+N-1}\,dr +H^{-kq} \right) \\&\quad \le C_1 M_\rho \left( A_H\right) \left( \frac{\sigma _N}{k+N}H^{1-q(k+N)} +H^{-kq} \right) , \end{aligned}$$

which conludes the proof in that case.

Case $-N<k\le 1-N$: In this case, we obtain from Proposition 2(ii) applied to $\rho _H$ the estimate

$$\begin{aligned} \int _{B_{A_H}}|x-y|^{k}\rho (y)\,dy\le C_2 M_\rho \left( A_H\right) T_k(|x|,A_H)|x|^{k} , \quad \forall x\in B_{A_H}^C, \end{aligned}$$

and integrating against $\rho (x)$ over $B_{A_H}^C$, we have

3.6 $$\begin{aligned} \int _{B_{A_H}^C}\int _{B_{A_H}}|x-y|^{k} \rho (x)\rho (y)\,dx\,dy\le C_2 M_\rho \left( A_H\right) \int _{B_{A_H}^C}T_k(|x|,A_H)|x|^{k} \rho (x)\,dx.\qquad \end{aligned}$$

We split the integral in the right hand side as $I_1+I_2$, where

$$\begin{aligned} I_1:=\int _{B_{A_H}^C\cap B_{H^{-q}}}T_k(|x|,A_H)|x|^{k} \rho (x)\,dx,\quad I_2:=\int _{B_{A_H}^C\setminus {B_{H^{-q}}}}T_k(|x|,A_H)|x|^{k} \rho (x)\,dx\, . \end{aligned}$$

Let us first consider $I_2$, where we have $|x|\ge H^{-q}\ge 2A_H$ on the integration domain. Since the map $|x|\mapsto \frac{|x|+A_H}{|x|-A_H}$ is monotonically decreasing to 1 in $(A_H,+\infty )$, it is bounded above by 3 on $(2A_H,+\infty )$. We conclude from (2.9) that $T_k(|x|,A_H)\le 3$ for $|x|\in (H^{-q},+\infty )$. This entails

3.7 $$\begin{aligned} I_2\le 3\int _{B_{A_H}^C\setminus {B_{H^{-q}}}}|x|^{k}\rho (x)\,dx\le 3\, H^{-kq}, \end{aligned}$$

where we used once again $|x|\ge H^{-q}$, recalling that $k<0$.

Concerning $I_1$, we have $\rho \le H$ on $B_{A_H}^C$ which entails

3.8 $$\begin{aligned} I_1\le H \int _{B_{A_H}^C\cap B_{H^{-q}}}T_k(|x|,A_H)|x|^{k} \,dx=\sigma _N H\int _{A_H}^{H^{-q}}T_k(r,A_H) r^{k+N-1}\,dr. \end{aligned}$$

If $-N<k<1-N$, we use (2.9) and $(r+2A_H)/(r+A_H)< 2$ for $r\in (0,+\infty )$, so that

3.9 $$\begin{aligned} \begin{aligned} \int _{A_H}^{H^{-q}}T_k(r,A_H) r^{k+N-1}\,dr&\le \int _{0}^{H^{-q}}\left( \frac{r+2A_H}{r+A_H}\right) ^{1-k-N} \,r^{k+N-1}\,dr \\&\le \frac{2^{1-k-N}}{k+N}\,H^{-q(k+N)}. \end{aligned} \end{aligned}$$

If $k=1-N$ we have from (2.9), since $2A_H\le H^{-q}<1$,

3.10 $$\begin{aligned} \begin{aligned} \int _{A_H}^{H^{-q}}T_k(r,A_H) r^{k+N-1}\,dr&=\int _{A_H}^{H^{-q}} \left( 1+\log \left( \frac{r+A_H}{r-A_H}\right) \right) \,dr\\&\le \int _{0}^{H^{-q}}\left( 1+\log \left( \frac{r+1}{r}\right) \right) \,dr\\&=H^{-q}+H^{-q}\log (1+H^q)+\log (1+H^{-q})\\ {}&\le H^{-q}(2+\log (1+H^q)). \end{aligned} \end{aligned}$$

Combining (3.8), (3.9), (3.10) we conclude $I_1\le \tfrac{\sigma _N2^{1-k+N}}{k+N}\, H^{1-q(k+N)}$ if $-N<k<1-N$, and $I_1\le \sigma _N H^{1-q}(2+\log (1+H^q))$ if $k=1-N$. These information together with the estimate (3.7) can be inserted into (3.6) to conclude. $\square$

We are now in a position to prove that any minimiser of ${{\mathcal {F}}}$ is bounded.

#### Theorem 7

Let $\chi >0$, $k \in (-N,0)$ and $m >m_c$. Then any global minimiser of ${{\mathcal {F}}}$ over ${{\mathcal {Y}}}$ is uniformly bounded and compactly supported.

#### Proof

Since $\rho$ is radially symmetric non-increasing by Proposition 4, it is enough to show $\rho (0)<\infty$. Let us reason by contradiction and assume that $\rho$ is unbounded at the origin. We will show that ${{\mathcal {F}}}[\rho ]-{{\mathcal {F}}}[{\tilde{\rho }}]>0$ for a suitably chosen competitor ${\tilde{\rho }}$,

$$\begin{aligned} {\tilde{\rho }}(x)={\tilde{\rho }}_{H,r}(x): = \frac{N M_\rho ({A_H})}{\sigma _N r^N} {\mathbb {1}}_{D_r}(x) + \rho (x) {\mathbb {1}}_{B_{A_H}^C}(x), \end{aligned}$$

where $B_{A_H}$ and *q* are defined as in Lemma 2, $B_{A_H}^C$ denotes the complement of $B_{A_H}$ and ${\mathbb {1}}_{D_r}$ is the characteristic function of a ball $D_r:=B_r(x_0)$ of radius $r>0$, centered at some $x_0\ne 0$ and such that $D_r\cap B_{A_H}=\emptyset$. Note that $A_H\le H^{-q}/2< H_0^{-q}/2< 1/2$. Hence, we can take $r>1$ and $D_r$ centered at the point $x_0=(2r,0, \dots , 0) \in {\mathbb {R}}^N$. Notice in particular that since $\rho$ is unbounded, for any $H>0$ we have that $B_{A_H}$ has non-empty interior. On the other hand, $B_{A_H}$ shrinks to the origin as $H\rightarrow \infty$ since $\rho$ is integrable.

As $D_r\subset B_{A_H}^C$ and $\rho ={\tilde{\rho }}$ on $B_{A_H}^C\setminus D_r$, we obtain

$$\begin{aligned} \begin{aligned} (m-1)\left( {{\mathcal {H}}}_m[\rho ]-{{\mathcal {H}}}_m[{\tilde{\rho }}]\right)&=\int _{B_{A_H}}\rho ^m\, dx + \int _{B_{A_H}^C} \rho ^m\, dx - \int _{B_{A_H}^C} \left( \rho +\frac{N M_\rho ({A_H})}{\sigma _N r^N}{\mathbb {1}}_{D_r}\right) ^m\, dx\\&=\int _{B_{A_H}}\rho ^m\, dx + \int _{D_r} \left[ \rho ^m - \left( \rho +\frac{N M_\rho ({A_H})}{\sigma _Nr^N}\right) ^m\right] \, dx\,. \end{aligned} \end{aligned}$$

We bound

$$\begin{aligned} \begin{aligned} \varepsilon _r:&=\int _{D_r} \left[ \rho ^m - \left( \rho +\frac{N M_\rho ({A_H})}{\sigma _Nr^N}\right) ^m\right] \, dx \le M_\rho (A_H)^m\left( \frac{\sigma _N}{N}\right) ^{1-m} r^{N(1-m)}, \end{aligned} \end{aligned}$$

where we use the convexity identity $(a+b)^m \ge \left| a^m-b^m\right|$ for $a,b >0$. Hence, $\varepsilon _r$ goes to 0 as $r\rightarrow \infty$. Summarising we have for any $r>1$,

3.11 $$\begin{aligned} (m-1)\left( {{\mathcal {H}}}_m[\rho ]-{{\mathcal {H}}}_m[{\tilde{\rho }}]\right) =\int _{B_{A_H}}\rho ^m\, dx+\varepsilon _r, \end{aligned}$$

with $\varepsilon _r$ vanishing as $r\rightarrow \infty$.

To estimate the interaction term, we split the double integral into three parts:

3.12 $$\begin{aligned} \begin{aligned} 2k\left( {{\mathcal {W}}}_k[\rho ]-{{\mathcal {W}}}_k[{\tilde{\rho }}]\right) =&\iint _{{\mathbb {R}}^N \times {\mathbb {R}}^N} |x-y|^{k}\left( \rho (x)\rho (y)-{\tilde{\rho }}(x){\tilde{\rho }}(y)\right) \,dxdy\\ =&\iint _{B_{A_H} \times B_{A_H}} |x-y|^{k}\rho (x)\rho (y)\,dxdy\\&+2\iint _{B_{A_H} \times B_{A_H}^C} |x-y|^{k}\rho (x)\rho (y)\,dxdy\\&+ \iint _{B_{A_H}^C \times B_{A_H}^C} |x-y|^{k}\left( \rho (x)\rho (y) - {\tilde{\rho }}(x){\tilde{\rho }}(y)\right) \,dxdy \\ =:&\,I_1+I_2+I_3(r)\, . \end{aligned} \end{aligned}$$

Let us start with $I_3$. By noticing once again that $\rho ={\tilde{\rho }}$ on $B_{A_H}^C\setminus D_r$ for any $r>0$, we have

$$\begin{aligned} I_3(r)=&\int \int _{D_r\times D_r}|x-y|^{k}\left( \rho (x)\rho (y)-{\tilde{\rho }}(x){\tilde{\rho }}(y)\right) \, dxdy\\&+2\int \int _{D_r\times (B_{A_H}^C\setminus D_r)}|x-y|^{k}\left( \rho (x)\rho (y)-{\tilde{\rho }}(x){\tilde{\rho }}(y)\right) \, dxdy\\ =:&\, I_{31}(r)+I_{32}(r)\, . \end{aligned}$$

Since ${\tilde{\rho }}=\rho +\frac{N M_\rho ({A_H})}{\sigma _N r^N}$ on $D_r$, we have

$$\begin{aligned} I_{32}(r)=-2\frac{N M_\rho ({A_H})}{\sigma _Nr^N}\int \int _{D_r\times (B_{A_H}^C\setminus D_r)}|x-y|^{k}\rho (y)\,dxdy\,. \end{aligned}$$

By the HLS inequality (3.1), we have

$$\begin{aligned} |I_{32}(r)|\le \,&2\frac{N M_\rho ({A_H})}{\sigma _Nr^N}\int \int _{D_r\times {\mathbb {R}}^N}|x-y|^{k}\rho (y)\,dxdy\\ \le \,&2C_{HLS}\frac{N M_\rho ({A_H})}{\sigma _Nr^N} \Vert {\mathbb {1}}_{D_r}\Vert _a\Vert \rho \Vert _{b} \end{aligned}$$

if $a>1, b>1$ and $1/a+1/b-k/N=2$. We can choose $b\in \left( 1,\min \left\{ m, \,N/(k+N)\right\} \right)$, which is possible as $-N<k<0, m>1$, and then we get $a>1$, $\rho \in L^b({\mathbb {R}}^N)$ as $1<b<m$, and

$$\begin{aligned} |I_{32}(r)|\le 2C_{HLS}||\rho ||_b M_\rho ({A_H})\,\left( \frac{\sigma _N r^N}{N}\right) ^{\frac{1}{a}-1}\, . \end{aligned}$$

The latter vanishes as $r\rightarrow \infty$. For the term $I_{31}$, we have

$$\begin{aligned} I_{31}(r)=&-2\frac{NM_\rho ({A_H})}{\sigma _Nr^N}\int \int _{D_r\times D_r}|x-y|^{k}\rho (y)\,dxdy\\&-\left( \frac{NM_\rho ({A_H})}{\sigma _Nr^N}\right) ^2\int \int _{D_r\times D_r}|x-y|^{k}\,dxdy\, . \end{aligned}$$

With the same choice of *a*, *b* as above, the HLS inequality implies

$$\begin{aligned} |I_{31}(r)|\le \,&2\frac{N M_\rho ({A_H})}{\sigma _Nr^N}\int \int _{D_r\times {\mathbb {R}}^N}|x-y|^{k}\rho (y)\,dxdy\\&+ \left( \frac{NM_\rho ({A_H})}{\sigma _Nr^N}\right) ^2\int \int _{D_r\times D_r}|x-y|^{k}\,dxdy\\ \le \,&C_{HLS}M_\rho ({A_H})\,\left( 2||\rho ||_b\left( \frac{\sigma _N r^N}{N}\right) ^{\frac{1}{a}-1}+ M_\rho ({A_H}) \left( \frac{\sigma _N r^N}{N}\right) ^{\frac{1}{a}+\frac{1}{b}-2}\right) , \end{aligned}$$

which vanishes as $r\rightarrow \infty$ since $a>1$ and $b>1$. We conclude that $I_3(r)\rightarrow 0$ as $r\rightarrow \infty$.

The integral $I_1$ can be estimated using Theorem 4, and the fact that $\rho \ge H>1$ on $B_{A_H}$ together with $m>m_c$,

3.13 $$\begin{aligned} I_1&= \iint _{B_{A_H} \times B_{A_H}} |x-y|^{k}\rho (x)\rho (y)\,dxdy \le C_* M_\rho (A_H)^{1+k/N} \int _{B_{A_H}}\rho ^{m_c}(x)\, dx \nonumber \\&\le C_* M_\rho (A_H)^{1+k/N} \int _{B_{A_H}}\rho ^{m}(x)\, dx\, . \end{aligned}$$

On the other hand, the HLS inequalities (3.1) and (3.2) do not seem to give a sharp enough estimate for the cross-term $I_2$, for which we instead invoke Lemma 2, yielding

3.14 $$\begin{aligned} I_2\le 2C_{k,N}\, M_{\rho }(A_H)\, {\mathcal {K}}_{k,q,N}(H), \end{aligned}$$

for given $q\in [0,m/N)$ and large enough *H* as specified in Lemma 2.

In order to conclude, we join together (3.11), (3.12), (3.13) and (3.14) to obtain for any $r>1$ and any large enough *H*,

3.15 $$\begin{aligned} {{\mathcal {F}}}[\rho ]-{{\mathcal {F}}}[{\tilde{\rho }}]&= {{\mathcal {H}}}_m[\rho ]-{{\mathcal {H}}}_m[{\tilde{\rho }}]+\chi \left( {{\mathcal {W}}}_k[\rho ]-{{\mathcal {W}}}_k[{\tilde{\rho }}]\right) \nonumber \\&\ge \left( \frac{1}{m-1} +\chi \frac{C_*}{2k} M_\rho ({A_H})^{1+k/N}\right) \,\int _{B_{A_H}}\rho ^{m}\, dx \nonumber \\&\quad +\chi \,\frac{C_{k,N}}{k}\, M_{\rho }({A_H})\, {\mathcal {K}}_{s,q,N}(H)\nonumber \\&\quad +\frac{\varepsilon _r}{m-1} +\frac{\chi }{2k} I_3(r)\,. \end{aligned}$$

Now we choose *q*. On the one hand, notice that for a choice $\eta >0$ small enough such that $m>m_c+\eta$, we have

3.16 $$\begin{aligned} \frac{2-m+\eta }{k+N}<\frac{m-1-\eta }{(-k)}\, . \end{aligned}$$

On the other hand, $-N<k<0$ implies $1-k/N>2N/(2N+k)$. Since $m>m_c$, this gives the inequality $m>2N/(2N+k)$. Hence, for small enough $\eta >0$ such that $m>N(2+\eta )/(2N+k)$, we have

3.17 $$\begin{aligned} \frac{2-m+\eta }{k+N}<\frac{m}{N}. \end{aligned}$$

Thanks to (3.16) and (3.17) we see that we can fix a non-negative *q* such that

3.18 $$\begin{aligned} \frac{2-m+\eta }{k+N}<q<\min \left\{ \frac{m}{N},\, \frac{(m-1-\eta )}{(-k)}\right\} . \end{aligned}$$

Since *q* satisfies (3.18), it follows that $-kq<m-1-\eta$ and at the same time $1-q(k+N)<m-1-\eta$, showing that ${\mathcal {K}}_{k,q,N}(H)$ from Lemma 2 grows slower than $H^{m-1-\eta }$ as $H\rightarrow \infty$ for $k \ne 1-N$. If $k=1-N$, we have that for any $C>0$ there exists $H>H_0$ large enough such that $CH^{1-q}\log (1+H^q)<H^{m-1-\eta }$ since $q>2-m+\eta$, and so the same result follows. Hence, for any large enough *H* we have

$$\begin{aligned} C_{k,N} \,M_{\rho }({A_H})\, {\mathcal {K}}_{k,q,N}(H)< C_{k,N} H^{m-1-\eta }\, M_\rho ({A_H})\le C_{k,N} H^{-\eta }\int _{B_{A_H}}\rho ^{m}\, dx \end{aligned}$$

since $\rho \ge H$ on $B_{A_H}$. Inserting the last two estimates in (3.15) we get for some $\eta >0$

$$\begin{aligned} {{\mathcal {F}}}[\rho ]-{{\mathcal {F}}}[{\tilde{\rho }}]&\ge \left( \frac{1}{m-1} +\chi \frac{C_*}{2k} M_\rho ({A_H})^{1+k/N} + \chi \,\frac{C_{k,N} H^{-\eta }}{k}\right) \,\int _{B_{A_H}}\rho ^{m}\, dx \\&\quad +\frac{\varepsilon _r}{m-1} +\frac{\chi }{2k} I_3(r)\,. \end{aligned}$$

for any $r>1$ and any large enough *H*. First of all, notice that $\int _{B_{A_H}}\rho ^{m}\, dx$ is strictly positive since we are assuming that $\rho$ is unbounded. We can therefore fix *H* large enough such that the constant in front of $\int _{B_{A_H}}\rho ^{m}$ is strictly positive. Secondly, we have already proven that $\varepsilon _r$ and $I_3(r)$ vanish as $r\rightarrow \infty$, so we can choose *r* large enough such that

$$\begin{aligned} {{\mathcal {F}}}[\rho ]-{{\mathcal {F}}}[{\tilde{\rho }}]>0. \end{aligned}$$

Let ${\tilde{{\tilde{\rho }}}}$ be defined by $\tilde{{\tilde{\rho }}}(x)={\tilde{\rho }}(x+{\tilde{x}})$, where ${\tilde{x}}=\int _{{\mathbb {R}}^N}x{\tilde{\rho }}(x)\,dx$. Since $\tilde{{\tilde{\rho }}}\in {\mathcal {Y}}$ and ${{\mathcal {F}}}[\tilde{{\tilde{\rho }}}\,]={{\mathcal {F}}}[{\tilde{\rho }}]$, we get a contradiction with the minimality of $\rho$. We conclude that minimisers of ${{\mathcal {F}}}$ over ${\mathcal {Y}}$ are bounded.

Finally, we can just use the Euler–Lagrange Eq. (3.5) and the same argument as for Corollary 2 to prove that $\rho$ is compactly supported. $\square$

### Regularity properties of global minimisers

This section is devoted to the regularity properties of global minimisers. With enough regularity, global minimisers satisfy the conditions of Definition 1, and are therefore stationary states of Eq. (1.1). This will allow us to complete the proof of Theorem 1.

We begin by introducing some notation and preliminary results. As we will make use of the Hölder regularising properties of the fractional Laplacian, see [48, 51], the notation

$$\begin{aligned} c_{N,s}(-{\varDelta })^s S_k[\rho ]=\rho \,, \quad s \in (0,N/2) \end{aligned}$$

is better adapted to the arguments that follow, fixing $s=(k+N)/2$, and we will therefore state the results in this section in terms of *s*.

One fractional regularity result that we will use repeatedly in this section follows directly from the HLS inequality (3.1) applied with $k=2s-N$: for any

$$\begin{aligned} s\in (0,N/2), \quad 1<p<\frac{N}{2s}, \quad q=\frac{Np}{N-2sp}, \end{aligned}$$

we have

3.19 $$\begin{aligned} (-{\varDelta })^s f \in L^p\left( {\mathbb {R}}^N\right) \; \Rightarrow \; f \in L^q\left( {\mathbb {R}}^N\right) \, . \end{aligned}$$

Further, for $1\le p<\infty$ and $s\ge 0$, we define the *Bessel potential space* ${\mathcal {L}}^{2s,p}({\mathbb {R}}^{N})$ as made by all functions $f\in L^{p}({\mathbb {R}}^{N})$ such that $(I-{\varDelta })^{s}f\in L^{p}({\mathbb {R}}^{N})$, meaning that *f* is the Bessel potential of an $L^{p}({\mathbb {R}}^N)$ function (see [52, pag. 135]). Since we are working with the operator $(-{\varDelta })^s$ instead of $(I-{\varDelta })^s$, we make use of a characterisation of the space ${\mathcal {L}}^{2s,p}({\mathbb {R}}^N)$ in terms of Riesz potentials. For $1<p<\infty$ and $0<s<1$ we have

3.20 $$\begin{aligned} {\mathcal {L}}^{2s,p}({\mathbb {R}}^{N})=\left\{ f\in L^{p}({\mathbb {R}}^{N}): f=g*W_{2s-N}, \; g\in L^{p}({\mathbb {R}}^{N})\right\} , \end{aligned}$$

see [49, Theorem 26.8, Theorem 27.3], see also Exercise 6.10 in Stein’s book [52, pag. 161]. Moreover, for $1\le p<\infty$ and $0<s<1/2$ we define the *fractional Sobolev* space ${{\mathcal {W}}}^{2s,p}({\mathbb {R}}^{N})$ by

$$\begin{aligned} {{\mathcal {W}}}^{2s,p}\left( {\mathbb {R}}^{N}\right) := \left\{ f \in L^{p}({\mathbb {R}}^{N}) : \iint _{{\mathbb {R}}^{N}\times {\mathbb {R}}^{N}}\frac{|f(x)-f(y)|^{p}}{|x-y|^{N+2s p}}dx\,dy <\infty \right\} . \end{aligned}$$

We have the embeddings

3.21 $$\begin{aligned}&{\mathcal {L}}^{2s,p}({\mathbb {R}}^N)\subset {{\mathcal {W}}}^{2s,p}({\mathbb {R}}^N)\quad \text{ for }\quad p\ge 2 ,\quad s\in (0,1/2), \end{aligned}$$

3.22 $$\begin{aligned}&{{\mathcal {W}}}^{2s,p}\left( {\mathbb {R}}^N\right) \subset C^{0,\beta }\left( {\mathbb {R}}^N\right) \quad \text{ for }\quad \beta = 2s-N/p,\quad p> N/2s, \nonumber \\&\quad s\in (0,1/2), \end{aligned}$$

see [52, pag. 155] and [22, Theorem 4.4.7] respectively.

Let $s\in (0,1)$ and $\alpha > 0$ such that $\alpha +2s$ is not an integer. Since $c_{N,s}(-{\varDelta })^sS_k[\rho ]=\rho$ holds in ${\mathbb {R}}^N$, then we have from [48, Theorem 1.1, Corollary 3.5] (see also [9, Proposition 5.2]) that

3.23 $$\begin{aligned} \Vert S_k[\rho ]\Vert _{C^{0,\alpha +2s}(\overline{B_{1/2}(0)})}\le c\left( \Vert S_k[\rho ]\Vert _{L^{\infty }({\mathbb {R}}^N)}+\Vert \rho \Vert _{C^{0,\alpha }(\overline{B_1(0)})}\right) \,, \end{aligned}$$

with the convention that if $\alpha \ge 1$ for any open set *U* in ${\mathbb {R}}^{N}$, then $C^{0,\alpha }({\overline{U}}):=C^{\alpha ',\alpha ''}({\overline{U}})$, where $\alpha '+\alpha ''=\alpha$, $\alpha ''\in [0,1)$ and $\alpha '$ is the greatest integer less than or equal to $\alpha$. With this notation, we have $C^{0,1}({\mathbb {R}}^N)=C^{1,0}({\mathbb {R}}^N)={{\mathcal {W}}}^{1,\infty }({\mathbb {R}}^N)$. In particular, using (3.23) it follows that for $\alpha > 0$, $s \in (0,1)$ and $\alpha +2s$ not an integer,

3.24 $$\begin{aligned} \Vert S_k[\rho ]\Vert _{C^{0,\alpha +2s}({\mathbb {R}}^{N})}\le c\left( \Vert S_k[\rho ]\Vert _{L^{\infty }({\mathbb {R}}^N)}+\Vert \rho \Vert _{C^{0,\alpha }({\mathbb {R}}^{N})}\right) \, . \end{aligned}$$

Moreover, rescaling inequality (3.23) in any ball $B_{R}(x_0)$ where $R\ne 1$ we have the estimate

3.25 $$\begin{aligned}&\sum _{\ell =0}^{\alpha _{2}}R^{\ell }\Vert D^{\ell } S_{k}[\rho ]\Vert _{L^{\infty }(B_{R/2}(x_{0}))}+ R^{\alpha +2s}[D^{\alpha _{1}}S_{k}[\rho ]]_{C^{0,\alpha +2s-\alpha _{2}}(B_{R/2}(x_{0}))} \nonumber \\&\quad \le C\left[ \Vert S_{k}[\rho ]\Vert _{L^{\infty }({\mathbb {R}}^N)}+ \sum _{\ell =0}^{\alpha _{1}}R^{2s+\ell }\Vert D^{\ell } \rho \Vert _{L^{\infty }(B_{R}(x_{0}))} +R^{\alpha +2s}[D^{\alpha _{1}}\rho ]_{C^{0,\alpha -\alpha _{1}}(B_{R}(x_{0}))} \right] \nonumber \\ \end{aligned}$$

where $\alpha _{1}, \alpha _{2}$ are the greatest integers less than $\alpha$ and $\alpha +2s$ respectively. In (3.25) the quantities $\Vert D^{\ell } S_{k}[\rho ]\Vert _{L^{\infty }}$ and $[D^{\ell }\rho ]_{C^{0,\alpha }}$ denote the sum of the $L^{\infty }$ norms and the $C^{0,\alpha }$ seminorms of the derivatives $D^{(\beta )} S_{k}[\rho ]$, $D^{(\beta )} \rho$ of order $\ell$ (that is $|\beta |=\ell$).

Finally, we recall the definition of $m_c$ and $m^*$ in (1.4) in terms of *s*: $m_c:=2-\frac{2s}{N}$ and

$$\begin{aligned} m^*:= {\left\{ \begin{array}{ll} \dfrac{2-2s}{1-2s}\, &{}\quad \text {if} \quad N\ge 1 \quad \text {and} \quad s \in (0,1/2), \\ + \, \infty &{}\quad \text {if} \quad N\ge 2 \quad \text {and} \quad s \in [1/2,N/2)\, . \end{array}\right. } \end{aligned}$$

Let us begin by showing that global minimisers of ${{\mathcal {F}}}$ enjoy the good Hölder regularity in the most singular range, as long as diffusion is not too slow.

#### Theorem 8

Let $\chi >0$ and $s \in (0,N/2)$. If $m_c<m< m^*$, then any global minimiser $\rho \in {{\mathcal {Y}}}$ of ${{\mathcal {F}}}$ satisfies $S_k[\rho ]=W_k*\rho \in {\mathcal {W}}^{1,\infty }({\mathbb {R}}^N)$, $\rho ^{m-1}\in {\mathcal {W}}^{1,\infty }({\mathbb {R}}^N)$ and $\rho \in C^{0,\alpha }({\mathbb {R}}^N)$ with $\alpha =\min \{1,\tfrac{1}{m-1}\}$.

#### Proof

Recall that the global minimiser $\rho \in {{\mathcal {Y}}}$ of ${{\mathcal {F}}}$ is radially symmetric non-increasing and compactly supported by Theorem 5 and Theorem 7. Since $\rho \in L^1\left( {\mathbb {R}}^N\right) \cap L^\infty \left( {\mathbb {R}}^N\right)$ by Theorem 7, we have $\rho \in L^p\left( {\mathbb {R}}^N\right)$ for any $1< p<\infty$. Since $\rho =c_{N,s}(-{\varDelta })^s S_k[\rho ]$, it follows from (3.19) that $S_k[\rho ]\in L^{q}({\mathbb {R}}^N)$, $q=\tfrac{Np}{N-2sp}$ for all $1<p<\tfrac{N}{2s}$, that is $S_k[\rho ]\in L^{p}({\mathbb {R}}^N)$ for all $p \in (\tfrac{N}{N-2s},\infty )$. Then, if $s\in (0,1)$, since $S_{k}$ is the Riesz potential of the density $\rho$ in $L^{p}$, by the characterisation (3.20) of the Bessel potential space, we conclude that $S_k[\rho ] \in {{\mathcal {L}}}^{2s,p}({\mathbb {R}}^N)$ for all $p>\tfrac{N}{N-2s}$. Let us first consider $s<1/2$, as the cases $1/2<s<N/2$ and $s=1/2$ follow as a corollary.

$0<s<1/2$: In this case, we have the embedding (3.21) and so $S_k[\rho ] \in {{\mathcal {W}}}^{2s,p}({\mathbb {R}}^N)$ for all $p\ge 2>\tfrac{N}{N-2s}$ if $N\ge 2$ and for all $p>\max \{2,\tfrac{1}{1-2s}\}$ if $N=1$. Using (3.22), we conclude that $S_k[\rho ] \in C^{0,\beta }\left( {\mathbb {R}}^N\right)$ with

$$\begin{aligned} \beta : = 2s-N/p, \end{aligned}$$

for any $p>\tfrac{N}{2s}> 2$ if $N\ge 2$ and for any $p>\max \{\tfrac{1}{2s},\tfrac{1}{1-2s}\}$ if $N=1$. Hence $\rho ^{m-1} \in C^{0,\beta }\left( {\mathbb {R}}^N\right)$ for the same choice of $\beta$ using the Euler–Lagrange condition (3.5) since $\rho ^{m-1}$ is the truncation of a function which is $S_k[\rho ]$ up to a constant.

Note that $m_c \in (1,2)$ and $m^*>2$. In what follows we split our analysis into the cases $m_c<m\le 2$ and $2<m < m^*$, still assuming $s<1/2$. If $m\le 2$, the argument follows along the lines of [12, Corollary 3.12] since $\rho ^{m-1} \in C^{0,\alpha }({\mathbb {R}}^N)$ implies that $\rho$ is in the same Hölder space for any $\alpha \in (0,1)$. Indeed, in such case we bootstrap in the following way. Let us fix $n \in {\mathbb {N}}$ such that

3.26 $$\begin{aligned} \frac{1}{n+1}<2s\le \frac{1}{n} \end{aligned}$$

and let us define

3.27 $$\begin{aligned} \beta _n:=\beta +(n-1)2s=2ns-N/p. \end{aligned}$$

Form (3.26) and (3.27) we see that by choosing large enough *p* there hold $1-2s<\beta _n<1$. Note that $S_k[\rho ] \in L^\infty \left( {\mathbb {R}}^N\right)$ by Lemma 1, and if $\rho \in C^{0,\gamma }\left( {\mathbb {R}}^N\right)$ for some $\gamma \in (0,1)$ such that $\gamma +2s<1$, then $S_k[\rho ] \in C^{0,\gamma +2s}\left( {\mathbb {R}}^N\right)$ by (3.24), implying $\rho ^{m-1} \in C^{0,\gamma +2s}\left( {\mathbb {R}}^N\right)$ using the Euler–Lagrange conditions (3.5). Therefore $\rho \in C^{0,\gamma +2s}\left( {\mathbb {R}}^N\right)$ since $m \in (m_c,2]$. Iterating this argument $(n-1)$ times starting with $\gamma =\beta$ gives $\rho \in C^{0,\beta _n}\left( {\mathbb {R}}^N\right)$ . Since $\beta _n<1$ and $\beta _n+2s>1$, a last application of (3.24) yields $S_k[\rho ]\in {\mathcal {W}}^{1,\infty }({\mathbb {R}}^N)$, so that $\rho ^{m-1}\in {\mathcal {W}}^{1,\infty }({\mathbb {R}}^N)$, thus $\rho \in {\mathcal {W}}^{1,\infty }({\mathbb {R}}^N)$. This concludes the proof in the case $m\le 2$.

Now, let us assume $2<m < m^*$ and $s<1/2$. Recall that $\rho ^{m-1}\in C^{0,\gamma }\left( {\mathbb {R}}^N\right)$ for any $\gamma <2s$, and so $\rho \in C^{0,\gamma }\left( {\mathbb {R}}^N\right)$ for any $\gamma <\tfrac{2s}{m-1}$. By (3.24) we get $S_k[\rho ]\in C^{0,\gamma }\left( {\mathbb {R}}^N\right)$ for any $\gamma <\tfrac{2s}{m-1}+2s$, and the same for $\rho ^{m-1}$ by the Euler–Lagrange equation (3.5). Once more with a bootstrap argument, we obtain improved Hölder regularity for $\rho ^{m-1}$. Indeed, since

3.28 $$\begin{aligned} \sum _{j=0}^{+\infty }\frac{2s}{(m-1)^j}=\frac{2s(m-1)}{m-2}\, \end{aligned}$$

and since $m < m^*$ means $\tfrac{2s(m-1)}{m-2} > 1$, after taking a suitably large number of iterations we get $S_k[\rho ]\in {\mathcal {W}}^{1,\infty }({\mathbb {R}}^N)$ and $\rho ^{m-1} \in {\mathcal {W}}^{1,\infty }({\mathbb {R}}^N)$. Hence, $\rho \in C^{0,{1}/{(m-1)}}\left( {\mathbb {R}}^N\right)$.

$N\ge 2$, $1/2\le s<N/2$: We start with the case $s=1/2$. We have $S_k[\rho ]\in L^p({\mathbb {R}}^N)$ for any $p>\tfrac{N}{N-1}$ as shown at the beginning of this proof. By (3.20) we get $S_k[\rho ] \in {{\mathcal {L}}}^{1,p}\left( {\mathbb {R}}^N\right)$ for all $p>\tfrac{N}{N-1}$. Then we also have $S_k[\rho ] \in {{\mathcal {L}}}^{2r,p}({\mathbb {R}}^N)$ for all $p>\tfrac{N}{N-1}$ and for all $r \in (0,1/2)$ by the embeddings between Bessel potential spaces, see [52, pag. 135]. Noting that $2\ge \tfrac{N}{N-1}$ for $N\ge 2$, by (3.21) and (3.22) we get $S_k[\rho ]\in C^{0,2r-N/p}({\mathbb {R}}^N)$ for any $r\in (0,1/2)$ and any $p>\tfrac{N}{2r}$. That is, $S_k[\rho ]\in C^{0,\gamma }({\mathbb {R}}^N)$ for any $\gamma \in (0,1)$. By the Euler–Lagrange Eq. (3.5), $\rho \in C^{0,\gamma \alpha }({\mathbb {R}}^N)$ with $\alpha =\min \{1,\tfrac{1}{m-1}\}$, and so (3.24) for $s=1/2$ implies $S_k[\rho ]\in {\mathcal {W}}^{1,\infty }({\mathbb {R}}^N)$. Again by the Euler–Lagrange Eq. (3.5), we obtain $\rho ^{m-1}\in {\mathcal {W}}^{1,\infty }({\mathbb {R}}^N)$.

If $1/2<s<N/2$ on the other hand, we obtain directly that $S_k[\rho ] \in {{\mathcal {W}}}^{1,\infty }({\mathbb {R}}^N)$ by Lemma 1, and so $\rho ^{m-1}\in {\mathcal {W}}^{1,\infty }({\mathbb {R}}^N)$.

We conclude that $\rho \in C^{0,\alpha }({\mathbb {R}}^N)$ with $\alpha =\min \{1,\tfrac{1}{m-1}\}$ for any $1/2\le s<N/2$. $\square$

#### Remark 2

If $m\ge m^*$ and $s<1/2$, we recover some Hölder regularity, but it is not enough to show that global minimisers of ${{\mathcal {F}}}$ are stationary states of (1.1). More precisely, $m \ge m^*$ means $\tfrac{2s(m-1)}{m-2} \le 1$, and so it follows from (3.28) that $\rho \in C^{0,\gamma }\left( {\mathbb {R}}^N\right)$ for any $\gamma <\tfrac{2s}{m-2}$. Note that $m \ge m^*$ also implies $\tfrac{2s}{m-2}\le 1-2s$, and we are therefore not able to go above the desired Hölder exponent $1-2s$.

#### Remark 3

In the arguments of Theorem 8 one could choose to directly bootstrap on fractional Sobolev spaces. In fact, for $0<s<1/2$ and $m > 2$ we have that $\rho ^{m-1}\in {{\mathcal {W}}}^{2s,p}({\mathbb {R}}^N)$ implies $\rho \in {{\mathcal {W}}}^{\frac{2s}{m-1},p(m-1)}({\mathbb {R}}^N)$. Indeed, let $\alpha <1$ and $u\in {{\mathcal {W}}}^{\alpha ,p}({\mathbb {R}}^N)$, where and $p\in [1,\infty )$. By the algebraic inequality $||a|^{\alpha }-|b|^{\alpha }|\le C|a-b|^{\alpha }$ we have

$$\begin{aligned} \iint _{{\mathbb {R}}^N\times {\mathbb {R}}^N}\frac{||u(x)|^{\alpha }-|u(y)|^{\alpha }|^{p/\alpha }}{|x-y|^{N+\alpha 2s(p/\alpha )}}\,dxdy\le c \iint _{{\mathbb {R}}^N\times {\mathbb {R}}^N}\frac{|u(x)-u(y)|^{p}}{|x-y|^{N+2sp}}\,dxdy\, , \end{aligned}$$

thus $|u|^{\alpha }\in {{\mathcal {W}}}^{\alpha s,p/\alpha }({\mathbb {R}}^N)$. This property is also valid for Sobolev spaces with integer order, see [41]. In particular, thanks to this property, in case $m\ge m^*$ we may obtain $\rho ^{m-1}\in {{\mathcal {W}}}^{\alpha ,p}({\mathbb {R}}^N)$ for any $\alpha <\frac{2s(m-1)}{m-2}$ and any large enough *p*, hence (3.22) implies that $\rho$ has the Hölder regularity stated in Remark 2.

We are now ready to show that global minimisers possess the good regularity properties to be stationary states of equation (1.1) according to Definition 1.

#### Theorem 9

Let $\chi >0$, $s \in (0,N/2)$ and $m_c<m < m^*$. Then all global minimisers of ${{\mathcal {F}}}$ in ${{\mathcal {Y}}}$ are stationary states of equation (1.1) according to Definition 1.

#### Proof

Note that $m<m^*$ means $1-2s<1/(m-1)$, and so thanks to Theorem 8, $S_k[\rho ]$ and $\rho$ satisfy the regularity conditions of Definition 1. Further, since $\rho ^{m-1} \in {\mathcal {W}}^{1,\infty }\left( {\mathbb {R}}^N\right)$, we can take gradients on both sides of the Euler–Lagrange condition (3.5). Multiplying by $\rho$ and writing $\rho \nabla \rho ^{m-1}=\tfrac{m-1}{m}\nabla \rho ^{m}$, we conclude that global minimisers of ${{\mathcal {F}}}$ in ${{\mathcal {Y}}}$ satisfy relation (2.1) for stationary states of Eq. (1.1). $\square$

In fact, we can show that global minimisers have even more regularity inside their support.

#### Theorem 10

Let $\chi >0$, $m_c<m$ and $s \in (0,N/2)$. If $\rho \in {{\mathcal {Y}}}$ is a global minimiser of ${{\mathcal {F}}}$, then $\rho$ is $C^\infty$ in the interior of its support.

#### Proof

By Theorem 8 and Remark 2, we have $\rho \in C^{0,\alpha }({\mathbb {R}}^N)$ for some $\alpha \in (0,1)$. Since $\rho$ is radially symmetric non-increasing, the interior of $\mathrm{supp\ }(\rho )$ is a ball centered at the origin, which we denote by *B*. Note also that $\rho \in L^1({\mathbb {R}}^N) \cap L^\infty ({\mathbb {R}}^N)$ by Theorem 7, and so $S_k[\rho ] \in L^\infty ({\mathbb {R}}^N)$ by Lemma 1.

Assume first that $s\in (0,1)\cap (0,N/2)$. Applying (3.25) with $B_R$ centered at a point within *B* and such that $B_R\subset \subset B$, we obtain $S_k[\rho ]\in C^{0,\gamma }(B_{R/2})$ for any $\gamma <\alpha +2s$. It follows from the Euler–Lagrange condition (3.5) that $\rho ^{m-1}$ has the same regularity as $S_k[\rho ]$ on $B_{R/2}$, and since $\rho$ is bounded away from zero on $B_{R/2}$, we conclude $\rho \in C^{0,\gamma }(B_{R/2})$ for any $\gamma <\alpha +2s$. Repeating the previous step now on $B_{R/2}$, we get the improved regularity $S_k[\rho ]\in C^{0,\gamma }(B_{R/4})$ for any $\gamma <\alpha +4s$ by (3.25), which we can again transfer onto $\rho$ using (3.5), obtaining $\rho \in C^{0,\gamma }(B_{R/4})$ for any $\gamma <\alpha +4s$. Iterating, any order $\ell$ of differentiability for $S_{k}$ (and then for $\rho$) can be reached in a neighborhood of the center of $B_R$. We notice that the argument can be applied starting from any point $x_{0}\in B$, and hence $\rho \in C^{\infty }(B)$.

When $N\ge 3$ and $s \in [1,N/2)$, we take numbers $s_1,\ldots , s_l$ such that $s_i\in (0,1)$ for any $i=1,\ldots , l$ and such that $\sum _{i=1}^l s_i=s$. We also let

$$\begin{aligned} S_k^{l+1}[\rho ]:=S_k[\rho ], \quad S_k^j[\rho ]:={\varPi }_{i=j}^l (-{\varDelta })^{s_j} S_k[\rho ], \quad \forall \, j \in \{1, \dots , l\}\, . \end{aligned}$$

Then $S_k^1[\rho ]=\rho$. Note that Lemma 1(i) can be restated as saying that $\rho \in {{\mathcal {Y}}}\cap L^\infty ({\mathbb {R}}^N)$ implies $(-{\varDelta })^{-\delta } \rho \in L^\infty ({\mathbb {R}}^N)$ for all $\delta \in (0,N/2)$. Taking $\delta =s-r$ for any $r \in (0,s)$, we have $(-{\varDelta })^{r}S_k[\rho ] =(-{\varDelta })^{r-s}\rho \in L^\infty$. In particular, this means $S_k^j[\rho ]\in L^\infty ({\mathbb {R}}^N)$ for any $j=1,\ldots , l+1$. Moreover, there holds

$$\begin{aligned} (-{\varDelta })^{s_j} S_k^{j+1}[\rho ]=S_k^j[\rho ], \quad \forall \, j \in \{1, \dots , l\}\, . \end{aligned}$$

Therefore we may recursively apply (3.25), starting from $S_k^1[\rho ]=\rho \in C^{0,\alpha }(B_R)$, where the ball $B_R$ is centered at a point within *B* such that $B_R\subset \subset B$, and using the iteration rule

$$\begin{aligned}&S_k^j[\rho ] \in C^{0,\gamma }(B_\sigma ) \; \Rightarrow \, S_k^{j+1}[\rho ] \in C^{0,\gamma +2s_j}\left( B_{\sigma /2}\right) \\&\quad \forall \, j \in \{1, \dots , l\}, \quad \forall \,\gamma >0 \text{ s.t. } \, \gamma +2s_j \, \text{ is } \text{ not } \text{ an } \text{ integer, }\, \quad \forall \, B_\sigma \subset \subset B. \end{aligned}$$

We obtain $S_{k}^{l+1}[\rho ]=S_k[\rho ]\in C^{0,\gamma }(B_{R/(2^l)})$ for any $\gamma <\alpha +2s$, and as before, the Euler–Lagrange Eq. (3.5) implies that $\rho \in C^{0,\gamma }(B_{R/(2^l)})$ for any $\gamma <\alpha +2s$. If we repeat the argument, we gain 2*s* in Hölder regularity for $\rho$ each time we divide the radius *R* by $2^l$. In this way, we can reach any differentiability exponent for $\rho$ around any point of *B*, and thus $\rho \in C^{\infty }(B)$. $\square$

#### Remark 4

We observe that the smoothness of minimisers in the interior of their support also holds in the fair-competition regime $m=m_c$. In such case global Hölder regularity was obtained in [12].

The main result Theorem 1 follows from Theorem 3, Corollary 2, Theorem 5, Proposition 4, Theorem 7, Theorem 9 and Theorem 10.

## Uniqueness

### Optimal transport tools

Optimal transport is a powerful tool for reducing functional inequalities onto pointwise inequalities. In other words, to pass from microscopic inequalities between particle locations to macroscopic inequalities involving densities. This sub-section summarises the main results of optimal transportation we will need in the one-dimensional setting. They were already used in [11] and in [13], where we refer for detailed proofs.

Let ${\tilde{\rho }}$ and $\rho$ be two probability densities. According to [7, 39], there exists a convex function $\psi$ whose gradient pushes forward the measure ${\tilde{\rho }}(a) da$ onto $\rho (x) dx$: $\psi '\# \left( {\tilde{\rho }}(a) da\right) = \rho (x) dx$. This convex function satisfies the Monge-Ampère equation in the weak sense: for any test function $\varphi \in C_b({\mathbb {R}})$, the following identity holds true

$$\begin{aligned} \int _{{\mathbb {R}}} \varphi (\psi '(a)) {\tilde{\rho }}(a)\, da = \int _{{\mathbb {R}}} \varphi (x) \rho (x)\, dx\, . \end{aligned}$$

The convex map is unique a.e. with respect to $\rho$ and it gives a way of interpolating measures using displacement convexity [40]. On the other hand, regularity of the transport map is a complicated matter. Here, as it was already done in [11, 13], we will only use the fact that $\psi ''(a) da$ can be decomposed in an absolute continuous part $\psi _{ac}''(a)da$ and a positive singular measure [53, Chapter 4]. In one dimension, the transport map $\psi '$ is a non-decreasing function, therefore it is differentiable a.e. and it has a countable number of jump singularities. For any measurable function *U*, bounded below such that $U(0) = 0$ we have [40]

4.1 $$\begin{aligned} \int _{{\mathbb {R}}} U({\tilde{\rho }}(x)) \, dx = \int _{{\mathbb {R}}} U\left( \dfrac{\rho (a)}{\psi _{ac}''(a)}\right) \psi _{ac}''(a)\, da\, . \end{aligned}$$

The following Lemma proved in [11] will be used to estimate the interaction contribution in the free energy.

#### Lemma 3

Let ${\mathcal {K}}:(0,\infty )\rightarrow {\mathbb {R}}$ be an increasing and strictly concave function. Then, for any $a, b \in {\mathbb {R}}$

4.2 $$\begin{aligned} {\mathcal {K}}\left( \frac{\psi '(b)-\psi '(a)}{b-a} \right) \ge \int _{0}^1 {\mathcal {K}} \left( \psi _{\mathrm {ac}}''([a,b]_s) \right) \, ds\, , \end{aligned}$$

where the convex combination of *a* and *b* is given by $[a,b]_s=(1-s)a+sb$. Equality is achieved in (4.2) if and only if the distributional derivative of the transport map $\psi ''$ is a constant function.

### Functional inequality in one dimension

In what follows, we will make use of a characterisation of stationary states based on some integral reformulation of the necessary condition stated in Proposition 4. This characterisation was also the key idea in [11, 13] to analyse the asymptotic stability of steady states and the functional inequalities behind.

#### Lemma 4

(Characterisation of stationary states) Let $N=1$, $\chi >0$ and $k\in (-1,0)$. If $m > m_c$ with $m_c=1-k$, then any stationary state ${\bar{\rho }} \in {{\mathcal {Y}}}$ of system (1.1) can be written in the form

4.3 $$\begin{aligned} {\bar{\rho }}(p)^m = \frac{\chi }{2} \int _{{\mathbb {R}}}\int _{0}^1 |q|^{k}{\bar{\rho }}(p -sq){\bar{\rho }}(p-sq+q)\, dsdq\, . \end{aligned}$$

The proof follows the same methodology as for the fair-competition regime [13, Lemma 2.8] and we omit it here.

#### Theorem 11

Let $N=1$, $\chi >0$, $k \in (-1,0)$ and $m>m_c$. If (1.1) admits a stationary density ${\bar{\rho }}$ in ${{\mathcal {Y}}}$, then

$$\begin{aligned} {{\mathcal {F}}}[\rho ]\ge {{\mathcal {F}}}[{\bar{\rho }}], \quad \forall \rho \in {{\mathcal {Y}}}\end{aligned}$$

with equality if and only if $\rho ={\bar{\rho }}$.

#### Proof

For a given stationary state ${\bar{\rho }} \in {{\mathcal {Y}}}$ and a given $\rho \in {{\mathcal {Y}}}$, we denote by $\psi$ the convex function whose gradient pushes forward the measure ${\bar{\rho }}(a) da$ onto $\rho (x) dx$: $\psi ' \# \left( {\bar{\rho }}(a) da\right) = \rho (x) dx$. Using (4.1), the functional ${{\mathcal {F}}}[\rho ]$ rewrites as follows:

$$\begin{aligned} {{\mathcal {F}}}[\rho ]&= \dfrac{1}{m-1}\int _{\mathbb {R}}\left( \dfrac{\bar{\rho }(a)}{\psi _{ac}''(a)}\right) ^{m-1} {\bar{\rho }}(a)\, da\\&\quad + \dfrac{\chi }{2k} \iint _{{\mathbb {R}}\times {\mathbb {R}}}\left| \dfrac{\psi '(a)-\psi '(b)}{a-b}\right| ^{k} |a-b|^{k} {\bar{\rho }}(a) {\bar{\rho }}(b) \, da db \\&= \dfrac{1}{m-1} \int _{\mathbb {R}}\left( \psi _{ac}''(a)\right) ^{1-m} \bar{\rho }(a)^m\, da\\&\quad + \dfrac{\chi }{2k} \iint _{{\mathbb {R}}\times {\mathbb {R}}} \big \langle \psi ''([a,b]) \big \rangle ^{k} |a-b|^{k} {\bar{\rho }}(a) {\bar{\rho }}(b) \, da db , \end{aligned}$$

where $\big \langle u([a,b]) \big \rangle = \int _0^1 u([a,b]_s)\, ds$ and $[a,b]_s=(1-s)a+bs$ for any $a,b \in {\mathbb {R}}$ and $u:{\mathbb {R}}\rightarrow {\mathbb {R}}_+$. By Lemma 4, we can write for any $a \in {\mathbb {R}}$,

$$\begin{aligned} (\psi _{ac}''(a))^{1-m} {\bar{\rho }}(a)^m = \frac{\chi }{2} \int _{{\mathbb {R}}} \big \langle \psi _{ac}''([a,b])^{1-m} \big \rangle |a-b|^{k}{\bar{\rho }}(a) {\bar{\rho }}(b) \, db, \end{aligned}$$

and hence

$$\begin{aligned} {{\mathcal {F}}}[\rho ] = \frac{\chi }{2} \iint _{{\mathbb {R}}\times {\mathbb {R}}} \left\{ \frac{1}{(m-1)} \big \langle \psi _{ac}''([a,b])^{1-m} \big \rangle +\frac{1}{k} \big \langle \psi ''([a,b]) \big \rangle ^{k}\right\} |a-b|^{k}{\bar{\rho }}(a) {\bar{\rho }}(b) \, da db\, . \end{aligned}$$

Using the concavity of the power function $(\cdot )^{1-m}$ and and Lemma 3, we deduce

$$\begin{aligned} {{\mathcal {F}}}[\rho ] \ge \frac{\chi }{2} \iint _{{\mathbb {R}}\times {\mathbb {R}}} \left\{ \frac{1}{(m-1)} \big \langle \psi ''([a,b]) \big \rangle ^{1-m} +\frac{1}{k} \big \langle \psi ''([a,b]) \big \rangle ^{k}\right\} |a-b|^{k}{\bar{\rho }}(a) {\bar{\rho }}(b) \, da db\, . \end{aligned}$$

Applying characterisation (4.3) to the energy of the stationary state ${\bar{\rho }}$, we obtain

$$\begin{aligned} {{\mathcal {F}}}[{\bar{\rho }}] = \frac{\chi }{2} \iint _{{\mathbb {R}}\times {\mathbb {R}}} \left( \frac{1}{(m-1)} +\frac{1}{k} \right) |a-b|^{k}{\bar{\rho }}(a) {\bar{\rho }}(b) \, da db\, . \end{aligned}$$

Since

4.4 $$\begin{aligned} \frac{z^{1-m}}{m-1}+\frac{z^k}{k} \ge \frac{1}{m-1}+\frac{1}{k} \end{aligned}$$

for any real $z>0$ and for $m>m_c=1-k$, we conclude ${{\mathcal {F}}}[\rho ]\ge {{\mathcal {F}}}[{\bar{\rho }}]$. Equality in (4.2) arises if and only if $\psi ''=1$, i.e. when $\rho ={\bar{\rho }}$. In agreement with this, equality in (4.4) is realised if and only if $z=1$. $\square$

In fact, the result in Theorem 11 implies that all critical points of ${{\mathcal {F}}}$ in ${{\mathcal {Y}}}$ are global minimisers. Further, we obtain the following uniqueness result:

#### Corollary 3

(Uniqueness) Let $\chi >0$ and $k \in (-1,0)$. If $m_c<m$, then there exists at most one stationary state in ${{\mathcal {Y}}}$ to equation (1.1). If $m_c<m<m^*$, then there exists a unique global minimiser for ${{\mathcal {F}}}$ in ${{\mathcal {Y}}}$.

#### Proof

Assume there are two stationary states to Eq. (1.1): ${\bar{\rho }}_1, {\bar{\rho }}_2 \in {{\mathcal {Y}}}$. Then Theorem 11 implies that ${{\mathcal {F}}}[{\bar{\rho }}_1]={{\mathcal {F}}}[{\bar{\rho }}_2]$, and so ${\bar{\rho }}_1$ is a dilation of ${\bar{\rho }}_2$. By Theorem 5, there exists a minimiser of ${{\mathcal {F}}}$ in ${{\mathcal {Y}}}$, which is a stationary state of Eq. (1.1) if $m_c<m<m^*$ by Theorem 9, and so uniqueness follows. $\square$

Theorem 11 and Corollary 3 complete the proof of the main result Theorem 2.

## Properties of the Riesz potential

The estimates in Proposition 2 are mainly based on the fact that the Riesz potential of a radial function can be expressed in terms of the hypergeometric function

$$\begin{aligned} F(a,b;c;z):=\frac{{\varGamma }(c)}{{\varGamma }(b){\varGamma }(c-b)}\int _{0}^1(1-zt)^{-a}(1-t)^{c-b-1}t^{b-1}\,dt, \end{aligned}$$

which we define for $z\in (-1,1)$, with the parameters *a*, *b*, *c* being positive. Notice that $F(a,b,c,0)=1$ and *F* is increasing with respect to $z\in (-1,1)$. Moreover, if $c>1$, $b>1$ and $c>a+b$, the limit as $z\uparrow 1$ is finite and it takes the value

A.1 $$\begin{aligned} \frac{{\varGamma }(c){\varGamma }(c-a-b)}{{\varGamma }(c-a){\varGamma }(c-b)}, \end{aligned}$$

see [34, §9.3]. We will also make use of some elementary relations. First of all, there holds

A.2 $$\begin{aligned} F(a,b;c;z)=(1-z)^{c-a-b} F(c-a,c-b;c;z), \end{aligned}$$

see [34, §9.5], and it is easily seen that

$$\begin{aligned} \frac{d}{dz}F(a,b;c;z)=\frac{ab}{c} F(a+1,b+1;c+1;z). \end{aligned}$$

Inserting (A.2) we find

A.3 $$\begin{aligned} \frac{d}{dz} F(a,b;c;z)=\frac{ab}{c} (1-z)^{c-a-b-1}F(c- a,c-b;c+1;z). \end{aligned}$$

To simplify notation, let us define

A.4 $$\begin{aligned} \begin{aligned} H(a,b;c;z)&:= \frac{{\varGamma }(b){\varGamma }(c-b)}{{\varGamma }(c)} F(a,b;c;z)\\&= \int _{0}^1(1-zt)^{-a}(1-t)^{c-b-1}t^{b-1}\,dt\, . \end{aligned} \end{aligned}$$

### Proof of Proposition 2

For a given radial function $\rho \in {{\mathcal {Y}}}$ we use polar coordinates, still denoting by $\rho$ the radial profile of $\rho$, and compute as in [50, Theorem 5], see also [1, 25] or [26, §1.3],

A.5 $$\begin{aligned} \begin{aligned}&|x|^k*\rho (x) \\&\quad = {\sigma _{N-1}} \int _0^{\infty } \left( \int _0^\pi \left( |x|^2+\eta ^2-2|x|\eta \cos \theta \right) ^{k/2}\,\sin ^{N-2} \!\theta \,d\theta \right) \,\rho (\eta ) \eta ^{N-1}\,d\eta \, . \end{aligned} \end{aligned}$$

Then we need to estimate the integral

A.6 $$\begin{aligned} {\varTheta }_k(r,\eta )&:= {\sigma _{N-1}} \int _0^\pi \left( r^2+\eta ^2-2r\eta \cos (\theta )\right) ^{k/2} \sin ^{N-2}(\theta )\, d\theta \nonumber \\&= {\left\{ \begin{array}{ll} r^k \vartheta _k\left( \eta /r\right) , &{}\quad \eta<r,\\ \eta ^k \vartheta _k\left( r/\eta \right) , &{}\quad r<\eta , \end{array}\right. } \end{aligned}$$

where, for $$u\in [0,1)$$,

$$\begin{aligned} \vartheta _k(u)&:= {\sigma _{N-1}} \int _0^\pi \left( 1+u^2-2u \cos (\theta )\right) ^{k/2} \sin ^{N-2}(\theta )\, d\theta \\&= {\sigma _{N-1}} \left( 1+u\right) ^k\int _0^\pi \left( 1-4\frac{u}{(1+u)^2} \cos ^2\left( \frac{\theta }{2}\right) \right) ^{k/2} \sin ^{N-2}(\theta )\, d\theta \, . \end{aligned}$$

Using the change of variables $t=\cos ^2\left( \frac{\theta }{2}\right)$, we get from the integral formulation (A.4),

A.7 $$\begin{aligned} \vartheta _k(u)&=2^{N-2} {\sigma _{N-1}} \left( 1+u\right) ^k\int _0^1 \left( 1-4\frac{u}{(1+u)^2}t\right) ^{k/2} t^{\frac{N-3}{2}} \left( 1-t\right) ^{\frac{N-3}{2}}\, dt\nonumber \\&= 2^{N-2} {\sigma _{N-1}} \left( 1+u\right) ^k H\left( a,b;c;z\right) \end{aligned}$$

with

$$\begin{aligned} a=-\frac{k}{2}, \quad b=\frac{N-1}{2}, \quad c=N-1, \quad z=\frac{4u}{(1+u)^2}\, . \end{aligned}$$

The function *F*(*a*, *b*; *c*; *z*) is increasing in *z* and then for any $z\in (0,1)$ there holds

A.8 $$\begin{aligned} F(a, b;c;z)\le \lim _{z\uparrow 1} F(a,b;c;z). \end{aligned}$$

Note that $c-a-b=(k+N-1)/2$ changes sign at $k=1-N$, and the estimate of ${\varTheta }_k$ depends on the sign of $c-a-b$:

Case $k>1-N$: The limit (A.8) is finite if $c-a-b>0$ and it is given by the expression (A.1). Therefore we get from (A.6), (A.7) and (A.4)

$$\begin{aligned} {\varTheta }_k(|x|,\eta )\le C_1(|x|+\eta )^{k}\le C_1|x|^{k}\quad \text {if} \, \, 1-N<k<0 \end{aligned}$$

with $C_1:= {2^{N-2}\sigma _{N-1}} {{\varGamma }(b){\varGamma }(c-a-b)}/{{\varGamma }(c-a)}$. Inserting this into (A.5) concludes the proof of (i).

Case $k < 1-N$: If $c-a-b<0$ we use (A.2)

$$\begin{aligned} F(a,b;c;z)=(1-z)^{c-a-b} F(c-a,c-b;c;z), \end{aligned}$$

where now the right hand side, using (A.8) and (A.1), can be bounded from above by $(1-z)^{c-a-b}{\varGamma }(c){\varGamma }(a+b-c)/[{{\varGamma }(a){\varGamma }(b)}]$ for $z\in (0,1)$. This yields from (A.6), (A.7) and (A.4) the estimate

A.9 $$\begin{aligned} {\varTheta }_k(|x|,\eta )\le C_2|x|^{k}\left( \frac{|x|+\eta }{|x|-\eta }\right) ^{1-k-N}\quad \text {if} \, \, k<1-N \end{aligned}$$

with $$C_2:= {2^{N-2}\sigma _{N-1}} {{\varGamma }(c-b){\varGamma }(a+b-c)}/{{\varGamma }(a)}$$.

Case $k=1-N$: If on the other hand $c-a-b=0$, we use (A.3) with $c=2a=2b=N-1$, integrating it and obtaining, since $F=1$ for $z=0$,

$$\begin{aligned} F(a,b;c;z)=1+\frac{N-1}{4}\int _{0}^z\frac{F(c-a,c-b;c+1;t)}{1-t}\,dt, \end{aligned}$$

and the latter right hand side is bounded above, thanks to (A.8) and (A.1), by

$$\begin{aligned} 1+\frac{(N-1){\varGamma }(N)}{4({\varGamma }(N/2+1/2))^2}\,\log {\left( \frac{1}{1-z}\right) } \end{aligned}$$

for $z\in (0,1)$. This leads from (A.6), (A.7) to the new estimate

A.10 $$\begin{aligned} {\varTheta }_k(|x|,\eta )\le C_2|x|^{k}\left( 1+\log \left( \frac{|x|+\eta }{|x|-\eta }\right) \right) \quad \text {if} \, \, k=1-N, \end{aligned}$$

with $$C_2:= 2^{N-2}\sigma _{N-1}\tfrac{{\varGamma }\left( N/2-1/2\right) ^2}{{\varGamma }(N-1)}\max \left\{ 1, \frac{(N-1){\varGamma }(N)}{2{\varGamma }\left( (N+1)/2\right) ^2}\right\} $$.

Now, if $\rho$ is supported on a ball $B_R$, the radial representation (A.5) reduces to

A.11 $$\begin{aligned} |x|^k *\rho (x) =\int _{0}^{R}{\varTheta }_k(|x|,\eta )\rho (\eta )\eta ^{N-1}\,d\eta ,\quad x\in {\mathbb {R}}^N. \end{aligned}$$

If $|x|>R$, we have $(|x|+\eta )(|x|-\eta )^{-1}\le (|x|+R)(|x|-R)^{-1}$ for any $\eta \in (0,R)$, therefore we can put *R* in place of $\eta$ in the right hand side of (A.9) and (A.10), insert into (A.11) and conclude. $\square$
